# Non-linear failure patterns in urban road networks exposed to flooding

**DOI:** 10.1371/journal.pone.0354204

**Published:** 2026-09-01

**Authors:** Konstantinos Karagiorgos, Nikos I. Kavallaris, Ole Sönnerborn, Fotis Maris, Konstantinos Spiliotis

**Affiliations:** 1 Risk and Environmental Studies, Karlstad University, Karlstad, Sweden; 2 Centre of Natural Hazards and Disaster Science (CNDS), Uppsala, Sweden; 3 Department of Mathematics and Computer Science, Karlstad University, Karlstad, Sweden; 4 Department of Civil Engineering, School of Engineering, Democritus University of Thrace, Xanthi, Greece; Shanghai University, CHINA

## Abstract

Urban road networks are highly sensitive to flooding, yet the systemic consequences of inundation on their structural and functional organization remain insufficiently understood. This study introduces a scale-independent analytical framework that integrates complex network measures such as degree, closeness, and betweenness centrality with community-structure analysis to quantify how flooding transforms network connectivity, accessibility, and mesoscale cohesion. This study assesses the impact of river flooding on the urban road network under two scenarios: a 100-year return period flood and the maximum probable scenario (BHF- Beräknat Högsta Flöde), which corresponds to an estimated extreme event with an approximate return period of 10,000 years. Our analysis shows that inundation under the two flood scenarios can generate non-linear and disproportionate impacts on connectivity, accessibility, and overall network cohesion. Specifically, our method: (a) localizes critically isolated nodes exposed to high risk; (b) identifies structural changes, showing that the flooded network is divided into three main large subnetworks with specific epicenters, along with 263 smaller subnetworks in the 100-year case and 553 in the BHF case; (c) estimates that network inefficiency, interpreted as analogous to mean travel time to a destination, increases by 120% and 170% for the 100-year and BHF scenarios, respectively; and (d) localizes critical roads that may serve as potential corridors during evacuation planning. These results offer actionable insights for disaster risk reduction, including planning for neighborhood-scale isolation, identifying vulnerable corridors, and designing redundant and decentralized emergency access routes. The framework is transferable to other urban areas where flood maps and road network data are available, supporting risk-informed spatial planning and strengthened civil protection.

## 1. Introduction

Infrastructure networks are fundamental to ensuring community accessibility and supporting sustainable economic activity [1]. An efficient network, comprising links, nodes, and transport services, facilitates the movement of people and goods, promotes trade, and strengthens social and regional cohesion [2]. However, the proper functioning of these networks is increasingly challenged by two major stressors: rapid population growth and destructive urban flooding [1, 3–5]. As climate change intensifies extreme rainfall and riverine flooding, both the frequency and severity of such events are expected to rise, placing additional pressure on urban transportation systems and the communities that depend on them [6]. Understanding the susceptibility of road networks to flooding is therefore essential, providing insights for stakeholders and policymakers seeking to enhance urban resilience [7].

Urban flooding can lead to road closures and force traffic to be diverted onto alternative routes, increasing the load on unaffected parts of the network. This added pressure may ultimately trigger congestion even on links far from the original flooded locations. Consequently, flooding and the resulting traffic congestion can substantially increase indirect failures, where road segments remain physically intact but become effectively disconnected from the rest of the transportation network [1].

A road network in transportation engineering is typically represented as a graph, where nodes correspond to intersections and links represent the road segments [8]. Road network vulnerability refers to the degree to which the network is susceptible to disruptions that lead to a reduction in network performance or serviceability [9]. Depending on the severity and location of a disruption, one or multiple road elements may lose functionality, altering the network’s topology and overall connectivity. A practical approach to quantify road network sensitivity is to evaluate the extent to which the network topology changes due to a disruption [7].

Previous research on flood–road network interactions has developed valuable, though still incomplete, mathematical perspectives on how transport systems fail under inundation. Casali and Heinimann [10] represent the road system as a primal graph and simulate flood scenarios by removing inundated links. They quantify impacts through changes in classical centrality measures (e.g., node and edge betweenness, closeness) and examine their spatial distributions across multiple return periods. Their findings reveal shifts in the location of critical nodes and corridors, yet the analysis largely treats centrality metrics independently and does not explicitly characterize how the network fragments into subnetworks or communities as failures accumulate. Fan *et al.* [11] adopt a different formalism by coupling a SEIR-type contagion model on edges with a percolation-based description of network degradation. Flood propagation, incubation, and recession are expressed through systems of ordinary differential equations whose solutions determine the time-varying proportion of flooded links and the evolution of connected components. Although this provides a rigorous temporal representation of failure dynamics, the approach primarily focuses on global percolation indicators and does not resolve how node-level accessibility and meso-scale structure evolve during inundation. Sant [12] applies a Multiple Centrality Assessment (MCA) framework in which several centrality indices (degree, closeness, betweenness, and occasionally straightness) are computed on flood-conditioned graphs to evaluate how pluvial inundation reshapes the spatial hierarchy of streets and the capacity of key corridors to sustain mobility. While this yields a multi-metric description of network change, it stops short of linking centrality shifts to non-linear patterns of fragmentation or community reorganisation. Wang *et al.* [13] further develop the percolation perspective by progressively removing flooded or low-quality links and quantifying robustness through order parameters such as the size of the giant component, critical thresholds, and areas under robustness curves. Their results reveal discontinuous, phase-transition-like losses of connectivity in large-scale road systems.

Despite these advances, existing approaches tend to emphasise either static topological metrics [10,12] or aggregate network-connectivity indicators [11,13], each providing only a partial view of how failures propagate through the system. Centrality-based studies quantify shifts in node importance but do not fully characterize how the network separates into functionally distinct subregions. Similarly, global connectivity-based approaches capture overall changes in network cohesion but provide limited insight into how accessibility and flow patterns reorganise at the node and meso-scale levels. To date, these complementary perspectives have rarely been integrated within a unified analytical framework for assessing flood-induced road-network evolution. The present work addresses this gap by jointly tracking degree, closeness, and betweenness centrality together with community-structure analysis across flood scenarios. This combination is essential because each metric captures a distinct dimension of network degradation: degree reflects the loss of local redundancy, closeness reveals the reorganization of accessibility, betweenness identifies emerging bottlenecks and flow concentration, and community detection exposes meso-scale fragmentation and the formation of isolated subnetworks. Analyzed jointly, these indicators reveal non-linear failure dynamics, such as the simultaneous emergence of uneven accessibility patterns and the concentration of connectivity onto critical corridors, which cannot be detected by any single metric applied in isolation.

This study applies the framework in a pilot setting to demonstrate its functionality while ensuring that it remains applicable to urban systems of different sizes and configurations. We begin by assessing the baseline structural properties of the urban network, highlighting key aspects of its organisation and inherent redundancy. Flood inundation data for multiple hazard scenarios are then used to identify the water depths and spatial extents that disrupt connectivity and render specific links or corridors inaccessible. The results reveal previously unrecognised critical nodes and subnetworks and show how the urban system reorganises under progressive disruption. In doing so, the framework provides actionable insights into the reconfiguration of urban road networks during flood events and offers practical support for civil protection planning and the design of strategies to mitigate flood-induced mobility loss.

## 2. Materials and methods

### 2.1. Study area

**Fig 1:**
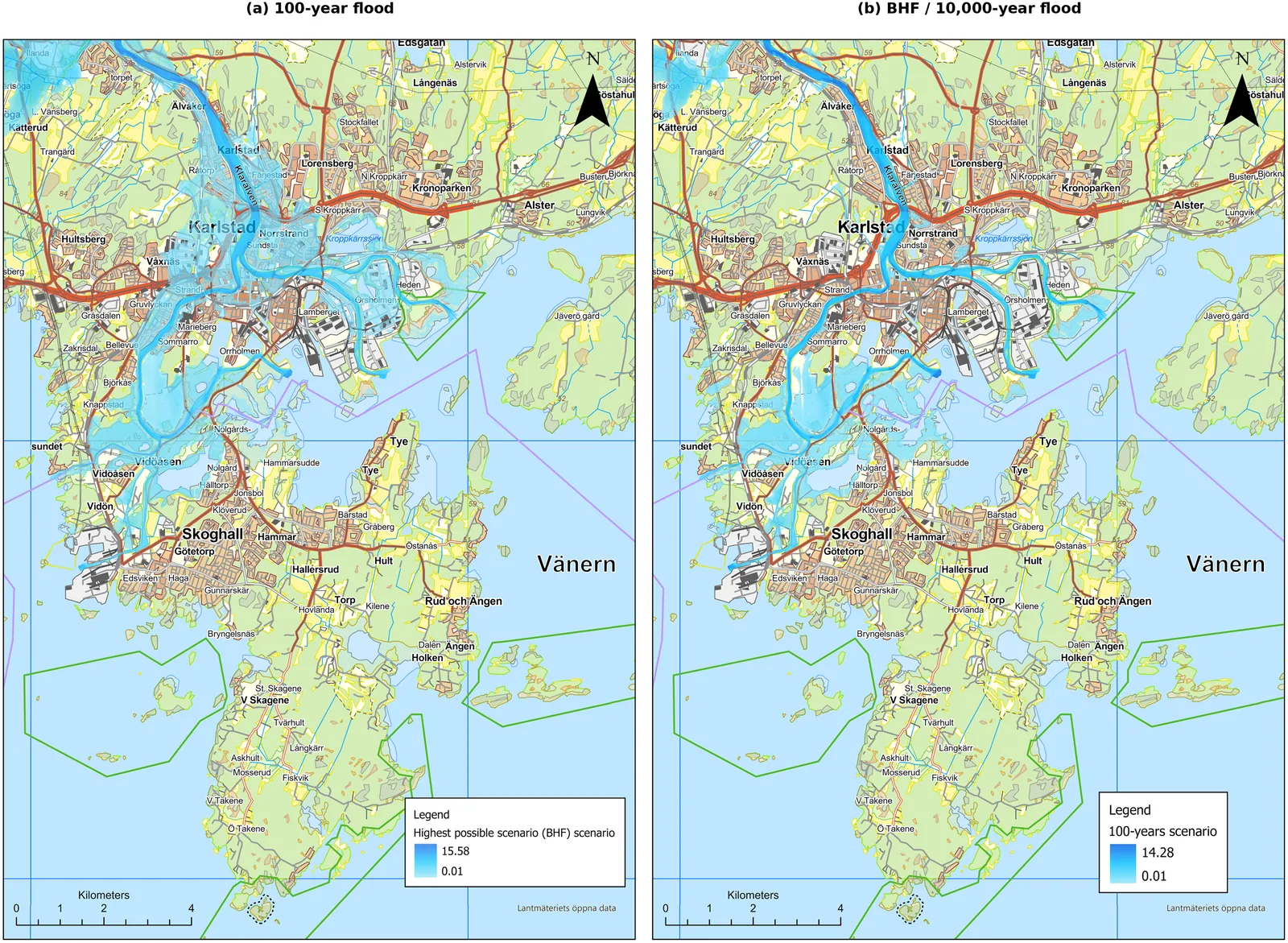
Flood inundation map of Karlstad city showing the study area. Inundation extent and water depth (m) in Karlstad, Sweden, under two probabilistic flood scenarios: (a) the 100-year return period flood, and (b) the maximum probable scenario (BHF – Beräknat Högsta Flöde), corresponding to an estimated extreme event with an approximate return period of 10,000 years. Inset: location of Karlstad within Sweden. Flood hazard data were obtained from the Swedish Civil Contingencies Agency (MSB), produced under the EU Floods Directive (2007/60/EC). The basemap is Sverigebaskarta – Vektor (vector tile service published by Esri Sverige, 2019), derived from Lantmäteriet’s open geodata products GSD-Sverigekartan, GSD-Vägkartan, and GSD-Terrängkartan, available under Creative Commons CC0. The map was produced by the authors using only the data sources listed above.

The city of Karlstad (Fig 1) is situated on the active river delta formed by the Klarälven River, where it discharges into Lake Vänern, one of Europe’s largest lakes. Hydrologically, Karlstad is exposed to flooding from two interacting sources: high discharges in the Klarälven and elevated water levels in Lake Vänern. Over the past century, Karlstad has experienced several major floods that have become reference events in policy and research on urban flood risk, notably those in 1987, 1995, and 2000–2001 [14]. The 2000–2001 event, in particular, was characterized by simultaneously high flows in the Klarälven and exceptionally high water levels in Lake Vänern, with lake levels remaining above critical thresholds for several months.

### 2.2. Data

#### 2.2.1. Flood hazard data.

The flood data sets used in this study were obtained from the Swedish Civil Contingency Agency (MSB). The flood hazard is represented by two return periods: the 100-year scenario and the maximum probable scenario (BHF – Beräknat Högsta Flöde), which corresponds to an estimated extreme event with an approximate return period of 10,000 years. The primary function of these maps was to comply with the requirements of the European Flood Risk Directive (Directive 2007/60), which established the foundation for national flood risk management and planning in Sweden. For the purpose of this study, the flood hazard layers were treated as inundation layers in the SWEREF99 coordinate system. The data sets are available to the public via MSB’s official flood mapping portal (https://gisapp.msb.se/Apps/oversvamningsportal/index.html).

#### 2.2.2. Road network.

The road network data utilised in this study were obtained from the GSD-Road Map dataset by the Swedish Mapping, Cadastral and Land Registration Authority [15]. The dataset offers a detailed and authoritative representation of Sweden’s national and regional road infrastructure, including attributes such as geometry, classification, and connectivity. It was originally developed to support spatial planning, infrastructure management, and mapping applications across the country. For this study, the section of the dataset covering the study area was extracted in the SWEREF99 coordinate system. The dataset is publicly available for scientific purposes through the authority’s official geodata portal.

### 2.3. Methods

#### 2.3.1. Representation of an urban network as a graph.

In this work, we represent the road network of Karlstad, Sweden, as a graph *G*, which enables the analysis of its structural and topological properties using tools from complex network theory. Formally, let *G* = (*V*, *E*), where *V* is the set of *nodes* (road intersections) and *E* is the set of *edges* (road segments connecting these intersections).

In the underlying urban road network, each intersection or crossroad with two-dimensional coordinates $v_{i}=(x_{i},y_{i})$ corresponds to a node $v_{i}\in V$. Two nodes $v_{i}$ and $v_{j}$ are connected by an edge $e_{ij}\in E$ if a road segment connects the corresponding intersections.

The *connectivity structure* of the graph is encoded in the *adjacency matrix* $A=[A_{ij}]$, whose elements are defined as


$$\begin{array}{c} A_{ij}=\left\{ \begin{array}{cc} 1, & \text{if an edge connects nodes }i\text{ and }j,\\ 0, & \text{otherwise.} \end{array}\right. \end{array}$$ (1)

Since each road has two lanes travelling in opposite directions, the road network is modelled as an undirected graph. This means that matrix *A* is symmetric: $A_{ij}=A_{ji}$.

**Fig 2:**
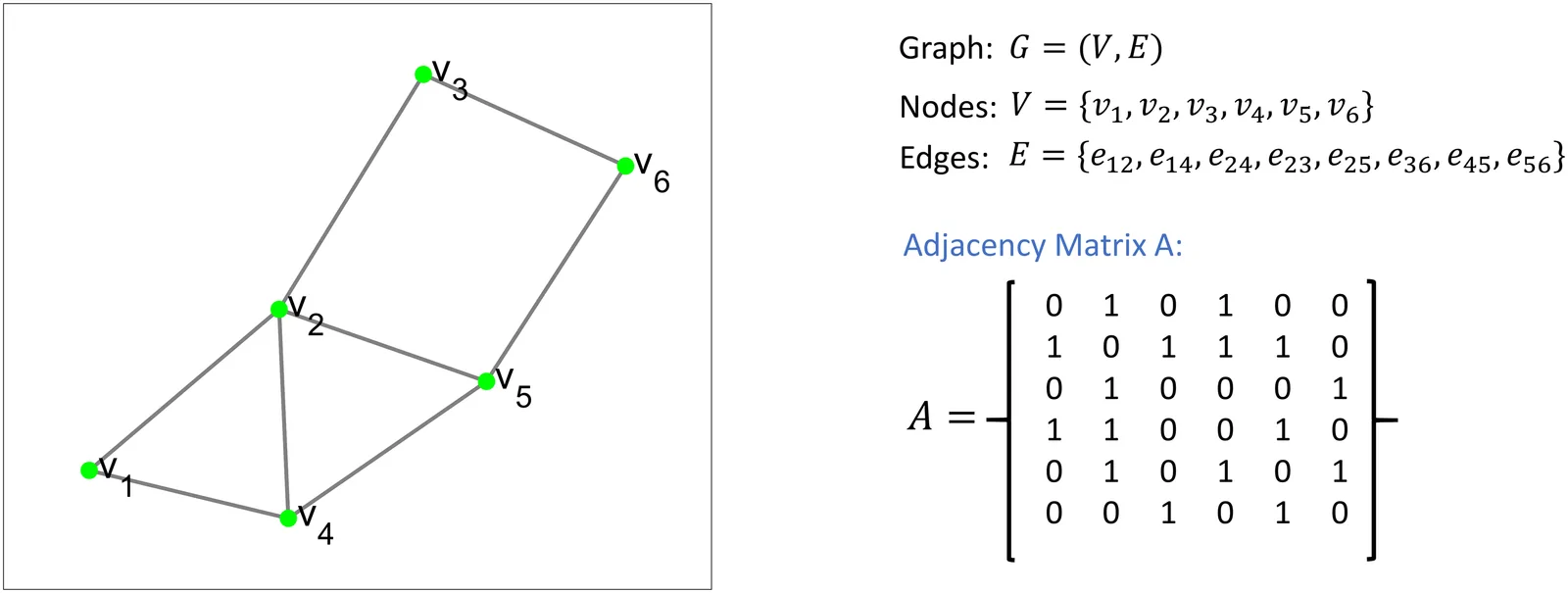
Graph representation of the road network, schematic example. A graph representing a road network with six crossroads and eight roads. The crossroads are represented by the set of nodes $V=\{v_{1},v_{2},v_{3},v_{4},v_{5},v_{6}\}$ and the roads by the set of edges $E=\{e_{12},e_{14},e_{23},e_{24},e_{25},e_{36},e_{45},e_{56},\}$. The connectivity structure is stored in the adjacency matrix *A*.

Fig 2 shows a six-node road network together with its adjacency matrix, which fully characterizes the connectivity structure of this simplified network.

#### 2.3.2. Modelling river flood-induced disruptions in an urban network.

During a flood event, substantial disruptions are expected to affect the functionality of the urban road network. The severity of these disruptions depends primarily on two factors:

(a) the intensity and extent of the flood,
(b) the structural configuration and connectivity of the network.

Flood intensity is represented through scenarios corresponding to different return periods—specifically, 100-year and 10,000-year events. These return periods indicate the statistical likelihood of a flood of a given magnitude occurring in any given year, corresponding respectively to annual exceedance probabilities of 1% and 0.01%. This range allows for the analysis of both typical and extreme flooding conditions. The flood extent and water depth data used in this study originate from the official flood hazard maps produced by the Swedish Civil Contingencies Agency (MSB). These nationwide hydrodynamic model outputs provide spatially distributed information on inundation extent and depth for each return period scenario. These model outputs are subsequently integrated with the road network to quantify flood-induced disruptions to connectivity and to assess the resulting impacts on overall network performance and resilience.

The data produced for the city of Karlstad by MSB can be organized in a set $W=\{(x_{k},y_{k},h_{k})\in {\mathbb{R}}^{3}\;|\;k=1,2,3,...,N\},$ where $\left(x_{k},y_{k}\right)$ represents spatial coordinates and $h_{k}$ the corresponding water height at $(x_{k},y_{k}).$ As expected, scenarios with higher return periods produce larger inundated areas, resulting in more extensive disruptions of the urban road network.

**Fig 3:**
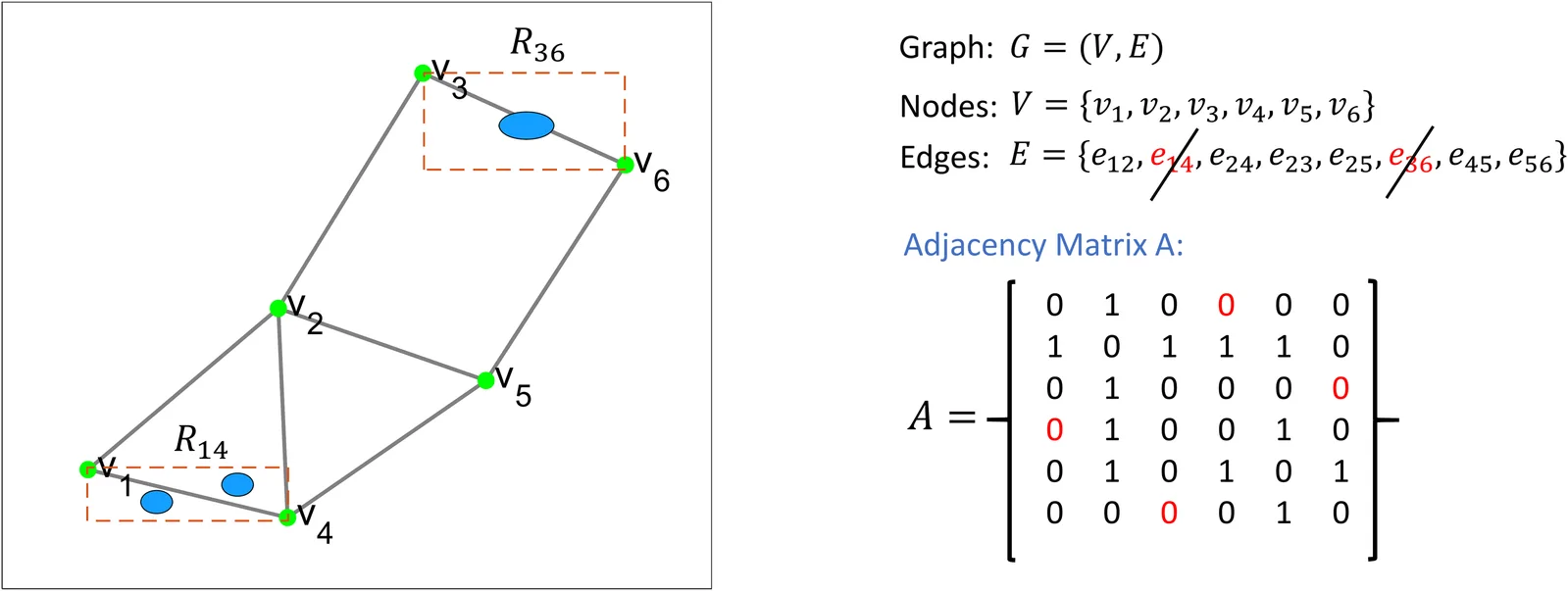
Flood-induced disruption of the urban road network. The rectangles *R*_14_ and *R*_36_ correspond to the areas surrounding edges *e*_14_ and *e*_36_, respectively. Both regions contain water points with depths exceeding 20 cm, leading to the removal of these edges from the network. Consequently, the associated entries in the adjacency matrix become zero ($A_{14}=A_{41}=A_{36}=A_{63}=0$), as indicated in red.

To model the impact of flooding on the road network, we apply the following procedure:

(1) For each edge $e_{ij}$ connecting nodes $v_{i}=(x_{i},y_{i})$ and $v_{j}=(x_{j},y_{j})$ we define a rectangular area
  $$\begin{array}{c} R_{ij}=\{(x,y)\in {\mathbb{R}}^{2}:\min\{x_{i},x_{j}\}<x<\max\{x_{i},x_{j}\},\;\min\{y_{i},y_{j}\}<y<\max\{y_{i},y_{j}\}\}. \end{array}$$
  This rectangle corresponds to the road area associated with edge $e_{ij}$, having the edge as its diagonal (see Fig 3).
(2) If any point $(x_{k},y_{k},h_{k})\in W$ within the rectangle $R_{ij}$ satisfies $h_{k}>0.20\;\text{m}$, the corresponding edge $e_{ij}$ is considered *flooded* and is therefore *removed* from the network topology. A road segment *k* was classified as flooded and removed from the functional road network when the inundation depth exceeded $h_{k}>0.2m.$ This threshold was adopted as an operational impassability criterion for regular passenger-vehicle traffic. Previous flood-transport studies indicate that shallow inundation can compromise vehicle movement, traction, and manoeuvrability, with reported disruption depths for passenger vehicles commonly falling within approximately 0.15–0.30 m depending on vehicle type, flow velocity, road surface, and driver behaviour [6,16]. The use of 0.2 m therefore provides a conservative and literature-supported criterion for identifying road segments that are functionally unavailable during flooding.

Steps 1 and 2 are repeated iteratively for all edges $e_{ij}\in E$ in the network to obtain the *post-flood network configuration*.

This procedure provides a systematic approach to translate spatial flood data into network-level disruptions, allowing for quantitative analysis of connectivity loss and network resilience under varying flood intensities.

A representative example of the proposed procedure is illustrated in Fig 3. In this example, the edges *e*_14_ and *e*_36_, along with their corresponding rectangular areas *R*_14_ and *R*_36_, are depicted. Both rectangles contain water points where the local water depth exceeds 20 cm. Consequently, the associated road segments *e*_14_ and *e*_36_ are considered flooded and are therefore removed from the network topology.

In the updated adjacency matrix, the elements corresponding to these edges are set to zero, i.e., $A_{14}=A_{41}=A_{36}=A_{63}=0,$ as indicated by the red-marked entries in Fig 3. The comparison between the pre-flood and post-flood network configurations allows the identification of nodes and areas that are most affected under the specific probabilistic hazard level considered. This analysis provides valuable insights into the spatial dependencies within the urban infrastructure and the potential cascade effects caused by localized flooding.

#### 2.3.3. Measures characterising urban network connectivity.

The next step in the methodology focuses on characterizing the network’s connectivity structure to identify critical nodes with high centrality and to analyze how the topology evolves under different flood return periods. This is accomplished using standard network metrics—degree centrality, mean path length, closeness centrality, and betweenness centrality—computed from the adjacency matrix, which encodes the complete topological information of the system [17,18].

These measures capture both local and global properties of the urban network. Local measures describe node-level features such as importance or influence within the network, while global measures summarize system-wide characteristics, including overall connectivity and efficiency. Statistical analyses of these metrics, such as their distributions and low-order statistics (mean and variance), provide aggregate descriptors that reveal large-scale structural and functional trends [17–19].

All computations are performed based on the adjacency matrix, which encodes the complete topological information of the network and serves as the foundation for deriving these centrality-based indicators.

#### 2.3.4. Degree centrality.

The *degree* of a node *i*, also referred to as its *degree centrality*, quantifies the number of edges directly connected to it [17]. For an undirected network, it is defined as:


$$\begin{array}{c} k_{i}=\sum_{j}A_{ij}, \end{array}$$ (2)

where $A_{ij}$ denotes the element of the adjacency matrix *A* ($A_{ij}=1$ if node *i* is connected to node *j*, otherwise $A_{ij}=0$).

A higher degree centrality indicates a node with stronger connectivity and, consequently, greater importance within the network. The *degree distribution*, *P*(*k*), describes the probability that a randomly selected node has degree *k*, thereby characterizing the overall connectivity pattern of the system.

#### 2.3.5. Mean Path Length and Closeness Centrality.

In a graph, a *path* is defined as a sequence of successive edges connecting a set of nodes, and its *length* corresponds to the number of edges traversed. Among all possible paths between two nodes, the ones with minimum length are referred to as *geodesic paths* or *shortest paths*. The length of a geodesic path (or the minimum length of the paths) connecting node *i* to *j* is denoted by $d_{i\to j}$.

By averaging the shortest path lengths over all pairs of nodes, we obtain the *mean path length* of the network [17,20]:


$$\begin{array}{c} \overset{―}{m}=\frac{\sum_{i,j}d_{i\to j}}{N(N-1)}, \end{array}$$

where *N* is the total number of nodes in the network. The mean path length reflects the network’s overall efficiency in transmitting flow. A smaller value of $\overset{―}{m}$ indicates that, on average, any two nodes can interact or exchange flow more rapidly.

In many cases, a network contains disconnected components—sets of nodes that are unreachable from the rest of the network. For such pairs of nodes, no path exists, so the path length is infinity. As a result, computing the mean path length over all node pairs becomes infeasible (infinity). To overcome this problem, we define the global efficiency as:


$$\begin{array}{c} E_{glob}=\frac{1}{N(N-1)}\sum_{i\neq j}\frac{1}{d_{i\to j}}. \end{array}$$

If no path exists, $d_{i\to j}=\infty$, and thus $\frac{1}{d_{ij}}=0$, allowing disconnected pairs to be handled. As an alternative to the mean path length, we define


$$\begin{array}{c} {\overset{―}{m}}_{2}=\frac{1}{E}. \end{array}$$ (3)

#### 2.3.6. Closeness centrality.

The concept of path length can also be applied locally to characterize the importance of individual nodes. Nodes that occupy central positions tend to have shorter average distances to all other nodes, indicating higher accessibility and influence within the network. This property is quantified by the *closeness centrality*, defined for node *i* as:


$$\begin{array}{c} Cl(i)=\frac{N-1}{\sum_{j}d_{i\to j}}, \end{array}$$ (4)

which represents the reciprocal of the average shortest path length from node *i* multiplied by the number of N−1 other nodes that can be reached from *i*. Nodes with higher closeness centrality values are more central, as they can reach other nodes through fewer steps, highlighting their key role in maintaining overall network connectivity and accessibility. In our computation, we normalized the closeness value dividing it by the maximum value of a network configuration.

#### 2.3.7. Betweenness centrality.

Another fundamental measure for assessing the importance of nodes within a network is the *betweenness centrality*. This metric quantifies the extent to which a node contributes to the overall flow of information by acting as an intermediary between other nodes. Nodes that serve as *bridges*—connecting distant or otherwise weakly linked parts of the network—typically exhibit high betweenness centrality values.

The betweenness centrality (BC) of a node *i* is defined as:


$$\begin{array}{c} BC(i)=\sum_{j\neq i\neq k}\frac{g_{jk}(i)}{g_{jk}}, \end{array}$$ (5)

where $g_{jk}(i)$ denotes the number of shortest paths between nodes *j* and *k* that pass through node *i*, and $g_{jk}$ is the total number of shortest paths between *j* and *k*. Thus, *BC*(*i*) represents the fraction of all shortest paths in the network that pass through node *i*, quantifying its role as a bridge or intermediary in the overall connectivity structure.

#### 2.3.8. Community structure and modularity analysis of an urban network.

In many real-world networks, nodes tend to form clusters or *communities*—subsets (subgraphs) characterized by dense internal connections and relatively sparse links to the rest of the network [19]. Identifying such modular structures is crucial for understanding how information, energy, or other flows are organized and processed within the system [21,22]. Community detection thus provides a modular view of the network, highlighting its functional organization.

From graph theory, the expected number of edges (or flow connections) between two nodes *i* and *j* in a random network with the same degree distribution is given by


$$\begin{array}{c} E=\frac{k_{i}k_{j}}{2m}, \end{array}$$

where $k_{i}$ and $k_{j}$ denote the degrees of nodes *i* and *j*, respectively (that is, the number of flow connections each node maintains) and $m=\frac{1}{2}\sum_{i}k_{i}$ is the total number of edges in the network [19,23]. The deviation of a subgraph *K* from this random configuration is quantified by


$$\begin{array}{c} Q_{1}(K)=\sum_{i,j\in K}\left(A_{ij}-\frac{k_{i}k_{j}}{2m}\right), \end{array}$$

which measures how strongly the flow connectivity within subgraph *K* differs from what would be expected by chance (e.g., in a configuration model network [24]).

Following [19], this concept is extended to define the *modularity* of an entire network. Let each node *i* be assigned to a community by an index $s_{i}$. For instance, in the case of two communities, we can set $s_{i}=\pm 1$. Then, the term $\frac{1}{2}(s_{i}s_{j}+1)$ equals 1 if nodes *i* and *j* belong to the same community and 0 otherwise. The modularity function is thus given by:


$$\begin{array}{c} Q_{2}=\frac{1}{4m}\sum_{i,j}\left(A_{ij}-\frac{k_{i}k_{j}}{2m}\right)(1+s_{i}s_{j})=\frac{1}{4m}\left[\sum_{i,j}\left(A_{ij}-\frac{k_{i}k_{j}}{2m}\right)+\sum_{i,j}\left(A_{ij}-\frac{k_{i}k_{j}}{2m}\right)s_{i}s_{j}\right]. \end{array}$$ (6)

The sum of all elements of the adjacency matrix *A* equals twice the total number of edges, i.e., $\sum_{ij}A_{ij}=\sum_{j}\sum_{i}A_{ij}=\sum_{j}k_{j}=2m$, which implies that the first part is equal to zero. Then, eq. (6) is written


$$\begin{array}{c} Q_{2}=\sum_{i,j}\left(A_{ij}-\frac{k_{i}k_{j}}{2m}\right)s_{i}s_{j}=\frac{1}{4m}{𝐬}^{T}B𝐬, \end{array}$$

where $𝐬=[s_{i}]$ and $B=[B_{ij}]$ with $B_{ij}=A_{ij}-\frac{k_{i}k_{j}}{2m}$ is the *modularity matrix*. The goal of community detection is to find the optimal partition of the network—that is, the assignment of nodes into subgraphs—that maximizes the modularity function *Q*_2_.

The modularity matrix *B* has a structure analogous to that of a graph Laplacian. Consequently, the optimization of *Q*_2_ can be efficiently performed using *graph partitioning* or *spectral partitioning* methods based on the eigenvalue–eigenvector decomposition of *B* [19,25].

## 3. Results

The flood hazard data described in Section 2 were used to assess the structural response of the urban road network under baseline conditions and two distinct flood scenarios. The modelling framework identifies flooded nodes and edges within the set *W*, thereby progressively reducing network connectivity as inundation thresholds are surpassed.

Three scenarios are analysed: (a) baseline conditions (no flooding), (b) a 100-year return period flood, and (c) the Beräknat Högsta Flöde (BHF) event, corresponding to an extreme flood with an approximate return period of 10,000 years. For each scenario, network properties and their relative variations are quantified to capture changes in system connectivity and performance. The urban network consists of 2,667 nodes (road intersections), while the number of edges decreases progressively with increasing flood intensity as inundated road segments become disconnected from the network.

### 3.1. Degree connectivity analysis

**Fig 4:**
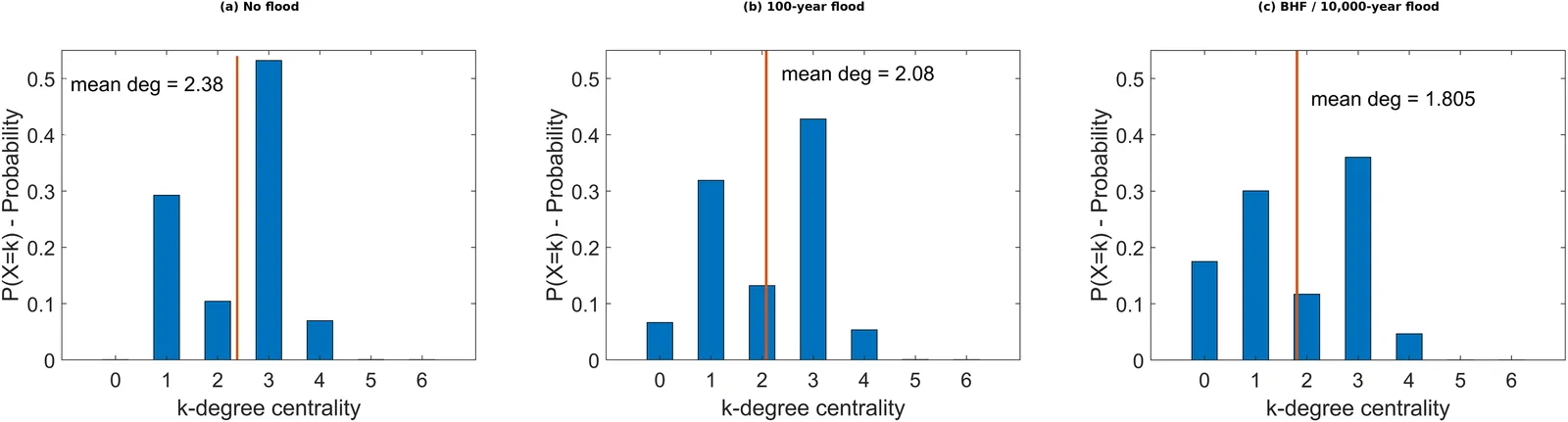
Degree distribution of the Karlstad road network under three flood scenarios. Red vertical line indicates the mean value of the degree distribution (a) no flooding, The majority of nodes have degree one and three, while there are no nodes with zero degree. (b) 100-year return period flood, and (c) the extreme flood scenario (BHF).

In order to assess the impact of flooding on the structural robustness of the urban transport system, we analyse the degree distribution of the urban network under the aforementioned three scenarios (Fig 4) inspired by [26,27]. Under baseline conditions (no flooding), the network exhibits a relatively homogeneous connectivity pattern, with most nodes having either one or three connections, and the maximum observed degree is six (Fig 4a). The corresponding mean degree is 2.382, indicating a well-connected urban structure with substantial local robustness and a small number of nodes with few or no connections. During the 100-year flood scenario, a clear reduction in network connectivity is observed. Several nodes become isolated (degree zero), and the proportion of nodes with only one or two connections increases. (Fig 4b). Although nodes with a degree of three remain common, the overall mean degree decreases to 2.086, driven by the loss of edges associated with inundated road segments. This indicates an early stage of fragmentation, where alternative routing options begin to diminish. Under the extreme flood scenario (BHF), this trend intensifies, with the number of isolated nodes nearly tripling relative to the baseline (Fig 4c), while the mean degree decreases more to 1.805, indicating a severe disruption to the network’s structural integrity. Overall, the degree connectivity distribution analysis results demonstrate that increasing flood intensity systematically decreases connectivity, as evidenced by a decline in the mean degree and an increase in the number of isolated nodes.

### 3.2. Closeness centrality analysis

**Fig 5:**
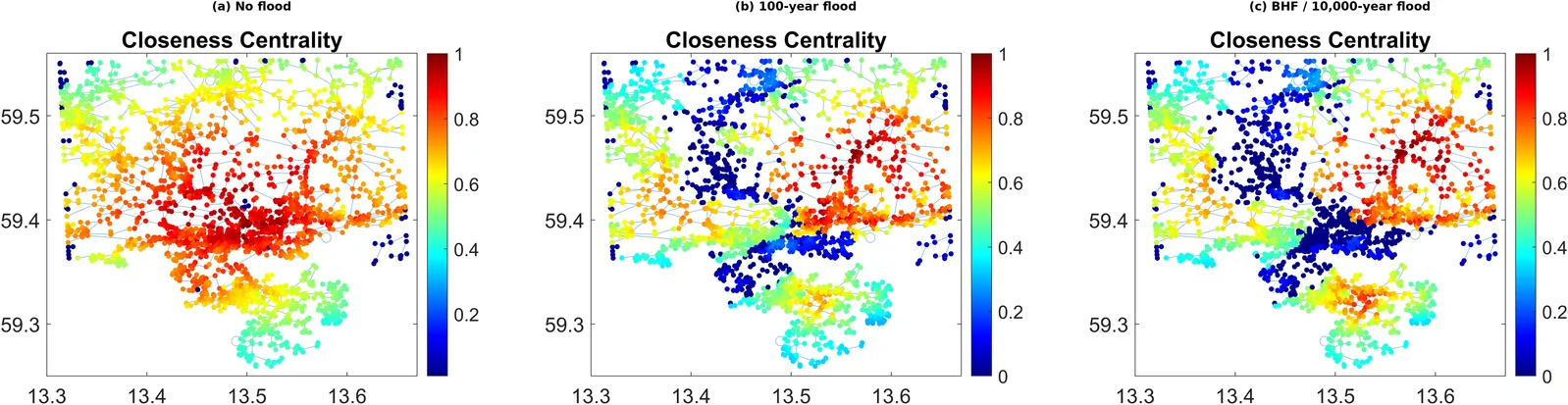
Closeness centrality of the road network under baseline and flood scenarios. In our computation, we normalized the closeness value, defined by (4), dividing it by the maximum value of the network configuration.. The color scale represents the magnitude of centrality values. (a) Baseline condition: the network exhibits a radially organized pattern of centrality centered around the urban core, reflecting a well-connected and resilient spatial structure. (b) 100-year flood scenario: the analysis reveals a distinct fragmentation of connectivity, dividing the network into three subregions—southern, northeastern, and northwestern—indicating reduced functional integration and isolated peripheral areas. (c) BHF flood scenario: while the overall topology remains broadly similar to the 100-year flood scenario (b), the spatial extent of inundation increases substantially, with flood-affected (blue-shaded) zones extending across major corridors. This results in a marked reduction of high-centrality nodes and a pronounced weakening of overall network cohesion, emphasising the system’s limited capacity to maintain accessibility under extreme flood conditions.

The influence of river flooding on the functionality of the urban road system is evaluated through a spatial analysis of closeness centrality, a measure that captures each node’s accessibility as the inverse of its cumulative shortest-path distance to all others, motivated by [10,28–31]. Nodes with high closeness centrality therefore represent locations that maintain efficient reachability within the network and play a disproportionate role in sustaining overall mobility. The spatial distribution of closeness values for the three scenarios is shown in Fig 5, where node colours reflect the magnitude of the centrality measure.

Under baseline (no flooding) conditions, the network exhibits a coherent and radially structured centrality pattern concentrated around the urban core (Fig 5a). This configuration is indicative of a well-integrated system in which most nodes can be reached with relatively short path lengths, reflecting both structural robustness and spatial efficiency. The 100-year flood scenario introduces a markedly different topology (Fig 5b). The inundation of low-lying corridors disrupts several key connectors, producing a clear fragmentation of the accessibility landscape. The network reorganises into three distinct subregions—southern, northeastern, and northwestern—with sharply reduced centrality in the boundary zones between them. This scenario, therefore, demonstrates that flooding under the 100-year return period is sufficient to induce spatially systematic declines in accessibility, rather than merely local disruptions. The BHF scenario amplifies these effects (Fig 5c). While the broader topology of the network remains recognisable, the extent of inundation increases substantially, removing additional edges that previously supported cross-regional connectivity. Flood-affected, low-centrality zones expand across major corridors, producing a widespread contraction of the high-centrality nodes that is more pronounced, indicating a significant weakening of network cohesion under extreme flooding.

**Fig 6:**
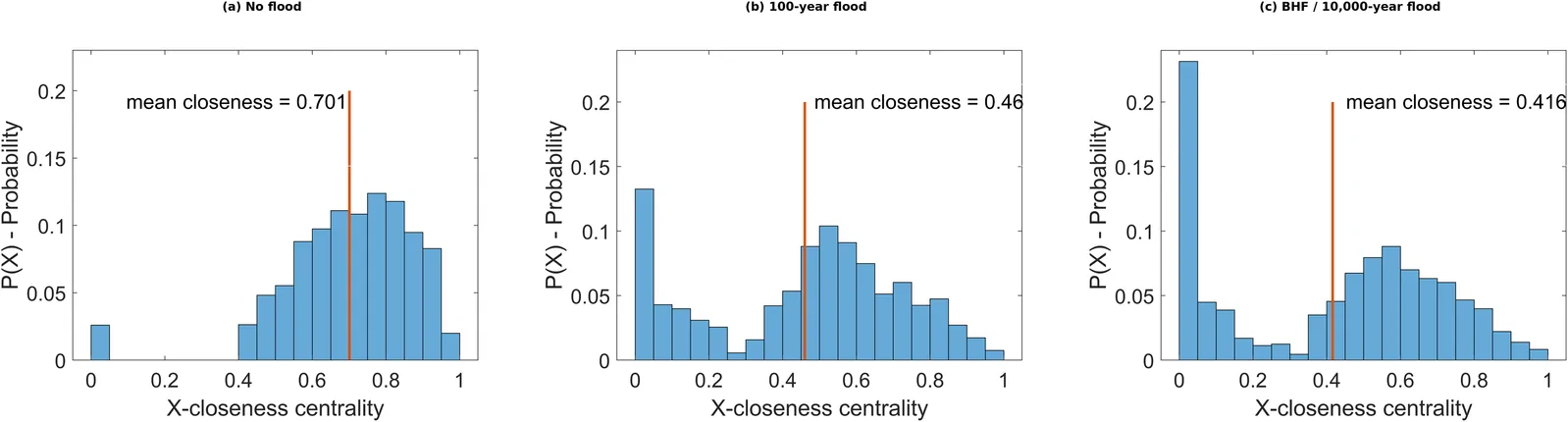
Distributions of closeness centrality under different flood scenarios. The corresponding means indicated with vertical lines. (a) In the baseline scenario, the distribution is nearly symmetric around its mean (vertical line mean = 0.701), reflecting a radial decrease in centrality from the urban core (Fig 5a). (b) Under the 100-year flood, the distribution becomes bimodal, with low values for inundated nodes and higher values for unaffected areas, indicating partial network segmentation. (c) In the BHF flood scenario, the bimodal pattern persists but with a wider inundation extent (Fig 5c), resulting in fewer high-centrality nodes and reduced network cohesion. The mean shortest path length is 36.52, implying an average of 37 steps between any two nodes.

To further characterise the effects of flooding in network accessibility, the spatial analysis is complemented by an examination of the centrality distributions, which provide a system-wide summary of the road network’s response under increasing flood severity (Fig 6). Under the baseline condition, the distribution in Fig 6a is approximately symmetric and unimodal, consistent with the radially decreasing centrality pattern observed in Fig 5a. This reflects a well-integrated system in which accessibility declines smoothly with distance from the urban core. The 100-year flood scenario produces a markedly different statistical pattern. As shown in Fig 6b, the distribution becomes distinctly bimodal, capturing the emergence of two dominant node classes: nodes within the inundated zone that exhibit very low centrality, and nodes in unaffected zones that retain substantially higher values. This pattern mirrors the spatial fragmentation reported in Fig 5b, where the network divides into three partially isolated subregions. In the BHF flood scenario, the bimodal structure persists but shifts further toward lower centrality values (Fig 6c). The expansion of inundation into major transport corridors (Fig 5c) eliminates several of the remaining high-centrality nodes and further reduces the coherence of the network. Compared with the 100-year event, the distribution is more heavily skewed toward low accessibility, underscoring the system’s diminishing capacity to support functional connectivity under extreme hydrological stress.

The mean shortest path length ${\overset{―}{m}}_{2}$ defined from eq.(3) increases across scenarios, from ${\overset{―}{m}}_{2}=37.7$ baseline to, ${\overset{―}{m}}_{2}=81.64$ in 100-year flood, and ${\overset{―}{m}}_{2}=102.08$ in BHF, reflecting a progressive deterioration in routing efficiency as inundation expands. This implies that, on average, two nodes in the network are connected through approximately 37 steps. The increase in this metric under flooding indicates a significant drop in routing efficiency and a weakening of overall network cohesion.

### 3.3. Betweenness centrality analysis

Betweenness centrality quantifies the importance of nodes that serve as intermediaries along the shortest paths between other nodes. These nodes effectively function as bridges connecting different parts of the urban network; consequently, major avenues or central roads typically exhibit high betweenness values, cf. [10,32–34].

**Fig 7:**
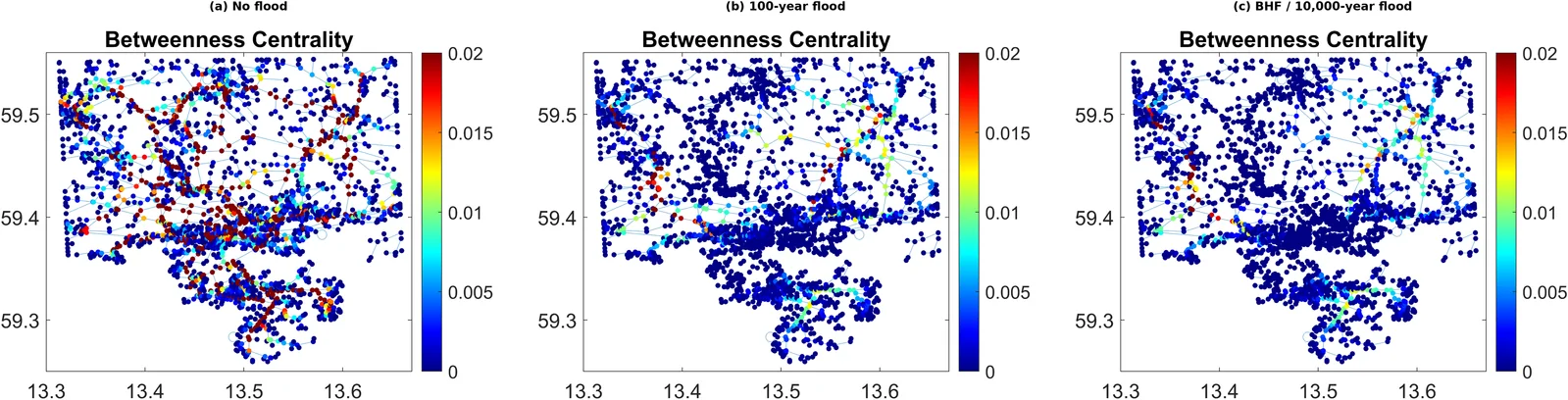
Spatial distribution of betweenness centrality (BC) values across the road network under three flood scenarios. The color scale represents the relative intensity of BC, with warmer colors indicating higher centrality. (a) Under baseline (no flooding) conditions, nodes with elevated BC values are primarily concentrated along the main arterial roads, reflecting their key role in overall connectivity. (b) During the 100-year flood scenario, nodes shift toward the primary routes that connect newly isolated or partially separated areas, indicating a reconfiguration of the network’s critical pathways. (c) Under the extreme BHF flood scenario, a limited number of nodes retain high BC values, signifying a substantial reduction in cross-regional connectivity and the emergence of a small set of bottleneck routes within an increasingly fragmented network.

Fig 7 depicts the spatial distribution of the evolving betweenness centrality (BC) values, leveraging equation (5), in the road network as the flooding scenarios progress. Under the baseline (no flooding) conditions, high BC values are concentrated along the main arterial and corridors of the city (red zones in Fig 7a), forming the structural backbone that supports efficient cross-city connectivity. During the 100-year flood scenario, several key routes are disrupted, leading to a spatial reorganisation of flow patterns. The BC spatial distribution reorganises into three principal corridors—northeastern, western, and southern (see Fig 7b)—reflecting the emergence of new critical pathways that maintain limited connectivity within the partially fragmented network, Fig 7b. Under the extreme BHF flood scenario, the number of high-BC nodes decreases sharply, with only a few remaining corridors retaining elevated centrality Fig 7c. These remaining routes act as bottlenecks within the network, indicating a transition from a robust, well-integrated system to a sparsely connected configuration with markedly reduced resilience and accessibility.

**Fig 8:**
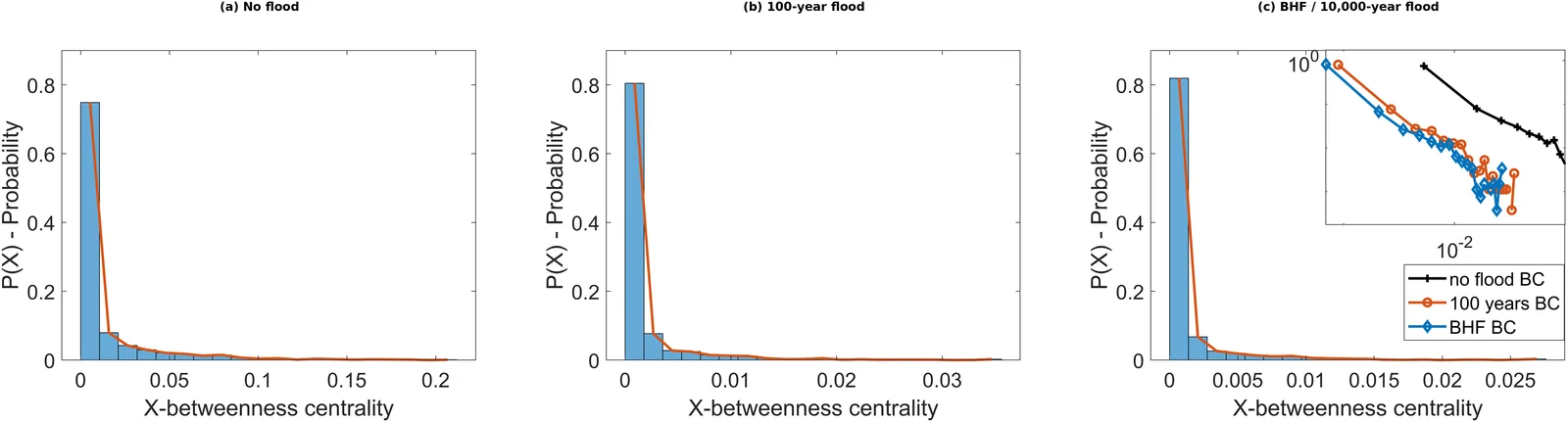
Betweenness centrality distributions for the three scenarios. In all cases, the distributions follow a power law of the form $P(x)=cx^{\alpha }$, with different values of the exponent α. (a) Baseline city network without flooding, α=−1.7101. (b) 100-year return-period flood scenario, α=−2.0759. (c) BHF flood scenario, α=−2.2.

Besides, Fig 8 presents the histogram of betweenness centrality (BC), which follows a power-law distribution of the form $P(x)=cx^{\alpha }$. For the no-flood scenario, the estimated critical exponent is α=−1.8856, with a 95% confidence interval of [−1.989,−1.7813]. Under the 100-year return period scenario, the distribution steepens to α=−2.0759, with a 95% confidence interval of [−2.1597,−1.99213]. For the BHF flodd scenario, the exponent further decreases to α=−2.2214, with a corresponding 95% confidence interval of [−2.3061,−2.1368].

The power-law behavior of betweenness centrality (BC) reflects the presence of a small number of structurally critical nodes—key intersections that channel a large share of shorter paths and thus exert disproportionate influence over the overall network flow. These nodes function as pivotal connectors within the urban road system, and their failure can severely compromise accessibility. Under the flood conditions, the estimated power-law exponent (α) becomes more negative, indicating an increasingly heterogeneous distribution of node importance. This steeper decay implies that a smaller subset of nodes carries disproportionately high centrality values, rendering the system more vulnerable to disruptions affecting these few critical points. Moreover, the changes observed in the distribution shape under extreme flooding (BHF scenario) suggest a broader reorganization of connectivity pathways, with flow redirected along a limited number of dominant routes. Collectively, the evolution of exponent (α) across scenarios may serve as a qualitative indicator flood severity, capturing the degree of topological imbalance and network fragmentation induced by escalating inundation levels.

### 3.4. Number of network components during flood

In the initial state (no flood), the city network is characterized by a giant component of 2,588 nodes, while there are a few small isolated components (22 clusters consisting of two nodes, 3 clusters of four nodes (with a tree structure), one cluster of six nodes, and one cluster of seven nodes).

To evaluate how flooding alters the community structure of the urban and road network, the modularity-based community detection method was applied to the post-network configurations. Under flood conditions, the removal of inundated links causes the network to break into multiple disconnected components, which can be interpreted as extreme forms of modularity where inter-community links have been entirely eliminated.

**Fig 9:**
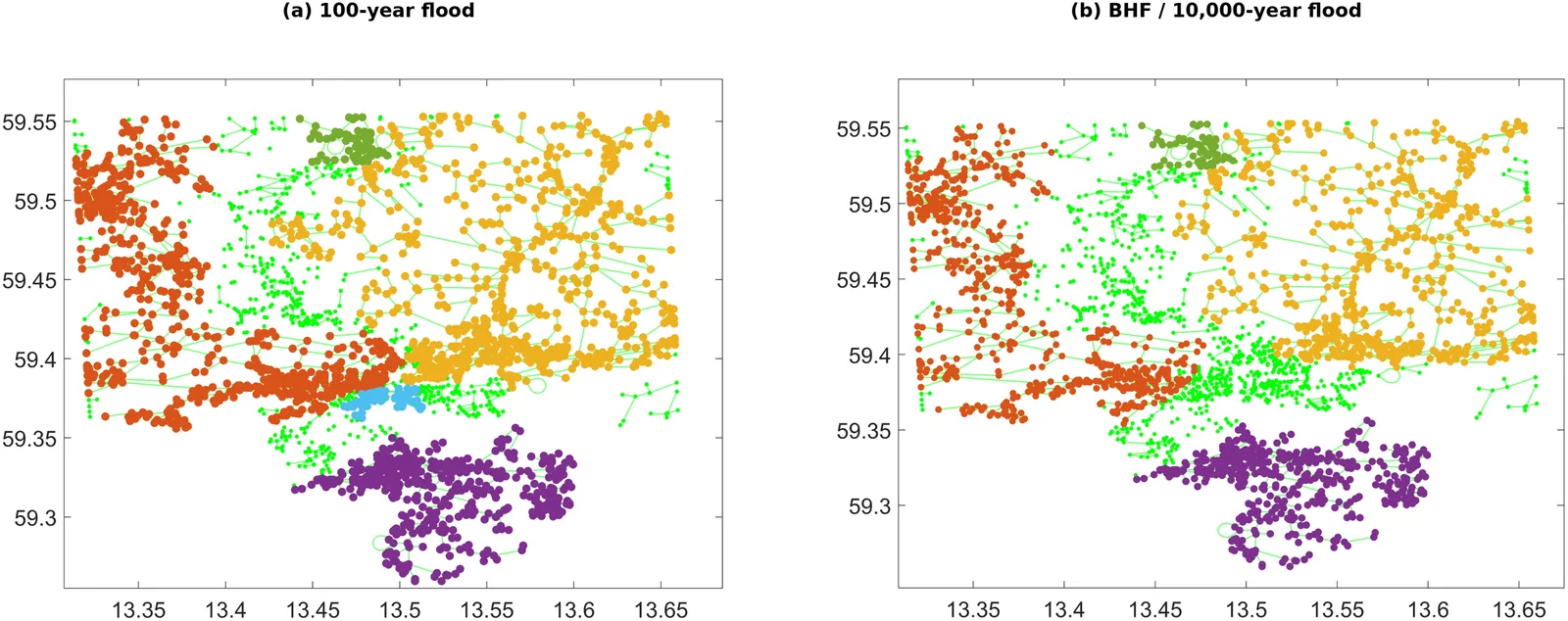
Disconnected components of the road network under (a) the 100-year return period flood scenario and (b) the BHF flood scenario. In the 100-year scenario, the network divides into five major components (each with more than 70 nodes), along with numerous smaller clusters and isolated nodes, indicating substantial fragmentation caused by the inundation of key bridging edges. Under the extreme BHF scenario, fragmentation intensifies: four large, spatially distinct components remain, with markedly fewer boundary connections between them. The number of isolated local clusters increases, reflecting widespread disconnection of low-lying and peripheral streets.

Fig 9 illustrates the resulting disconnected components for the two severe flood scenarios. In the 100-year flood scenario (Fig 9a), the network is partitioned into five major components, each containing more than 70 nodes. Specifically, the network contains three major components comprising 716 (red), 744 (yellow), and 463 (purple) nodes, respectively (Fig 9a). In addition, 263 smaller components have become mutually disconnected following the flooding of key bridging edges. The majority of these 263 smaller clusters are isolated nodes — shown in light green — mainly located near the river, indicating the fragmentation of local streets and secondary connections. Under the extreme BHF scenario (Fig 9b), only four large components with more than 70 nodes remain, each corresponding to a major subregion of the city. At the same time, the number of small isolated components increases from 263 in the 100-year scenario to 553. Specifically, the network now contains three major components comprising 625 (red), 619 (yellow), and 465 (purple) nodes, respectively, as shown in Fig 9b. This indicates a substantial reduction in the number of nodes in the major components compared with the 100-year scenario. Considering both the increase in isolated nodes, defined as nodes with degree zero (Fig 4c), and the rise in the number of isolated clusters from 264 to 553, we conclude that network fragmentation becomes more severe under the BHF scenario. The light green cluster of isolated nodes becomes more pronounced (compared to the 100-year case), reflecting widespread disconnection of local roads, especially in low-lying and peripheral areas.

Overall, the component structure under both scenarios demonstrates a substantial collapse of network-wide cohesion. The progressive increase in the number and spatial extent of disconnected components aligns with the modularity framework, where severe flooding effectively maximizes separation between subgraphs, leaving only internally dense but mutually isolated clusters of the urban network.

## 4. Discussion

This study demonstrates how river flooding can trigger both structural and functional reorganization within urban road networks. By integrating high-resolution flood hazard information with complex network analysis, we capture non-linear alterations in network connectivity, accessibility, and overall systemic cohesion. In particular, we employ degree analysis, closeness centrality, betweenness centrality, and network component analysis to quantify how inundation affects the configuration and performance of the road network. The resulting patterns show how hydrological disturbances propagate through the urban mobility system, producing cascading impacts that are directly relevant to flood risk assessment, disaster risk reduction, and civil protection planning.

The degree connectivity analysis shows that already under the 100-year scenario, the network experiences clear signs of structural degradation (Fig 4). The reduction in mean degree and the increasing number of isolated nodes indicate a loss of redundancy and an early stage of fragmentation. This network behaviour aligns with findings from Dong *et al.* [1] who demonstrated that even modest flood-induced disruptions can produce disproportionately large reductions in network connectivity. Our results show that a similar sensitivity emerges in smaller-scale systems like Karlstad municipality, where limited redundancy along key corridors allows flooding under the 100-year return period to significantly constrain routing options. The progressive loss of degree-connected nodes (BHF scenario) translates into fewer alternative routes available during emergency operations. This structural degradation constrains evacuation mobility, slows access for emergency services, and increases the likelihood of local isolation—particularly in low-lying neighbourhoods.

Closeness centrality captures how accessibility reorganises under flood scenarios, cf. [28–31]. While the baseline network exhibits a radially integrated structure centered around the urban core, both flood scenarios fragment this into spatially distinct accessibility regions. The 100-year flood divides the system into three functional subregions, and the BHF event intensifies the fragmentation by reducing high-centrality zones. The emergence of bimodal closeness distributions highlights that the system does not degrade gradually; instead, accessibility reorganises in a non-linear fashion. Such abrupt transitions are consistent with the discontinuous failure behaviour described by Wang *et al.* [3], who stated that local inundation can trigger large-scale systemic collapse in road networks when critical nodes are removed. Another valuable insight provided by closeness centrality concerns the post-organization structure of the city network. As shown in Fig 5b and 5c, the structure of the initial network changes substantially under flooding. The flooded river broadly divides the city network into three main areas: east, west, and south of the river. The red regions in Fig 5b indicate the epicentres of these subnetworks. This spatial separation persists under the more intense BHF scenario (Fig 5c). The same pattern is further supported by the component analysis, which identifies three major components. From a civil protection perspective, response efforts should be reorganized around these new epicenters, for example, by locating emergency facilities and critical resources in their vicinity (healthcare facilities, police, rescue utilities, etc.).

Betweenness centrality reveals how shortest-path flows become increasingly concentrated as flooding progresses, cf. [32–34]. Flooding redistributes flow onto fewer, more critical connectors, while the steepening of the betweenness distribution indicates growing dependency on a small subset of nodes. Under the BHF scenario, only a limited number of corridors retain high structural importance, forming bottlenecks that would sever the remaining cross-regional connectivity if disrupted. These changes correspond with earlier observations by Casali and Heinimann [10], who found that flooding alters the spatial distribution of critical nodes, with some roads gaining or losing importance depending on inundation patterns. From a risk analysis perspective, this highlights that a road network’s vulnerability cannot be inferred from the baseline topology only; its hazard-conditioned configuration must also be assessed.

The community analysis shows that river flooding reorganises the road system into distinct structural components that behave as semi-independent subnetworks. Under the 100-year flood scenario, five major components emerge, whereas the BHF scenario produces four, larger but more spatially isolated regions. This progressive loss of a single, city-wide giant component and the emergence of multiple disconnected clusters is consistent with fragmentation patterns reported in percolation-based studies of flooded road networks [3,26,35]. Compared with previous work that has focused primarily on global robustness indicators, percolation thresholds, and centrality-based vulnerability metrics [3,10], our explicit use of community detection provides a complementary perspective on how flood-induced failures partition the network into functional “islands” of connectivity. In this sense, the application of community-structure analysis to fluvial flood scenarios at the intra-urban scale remains relatively novel, extending existing approaches by capturing the meso-scale organization of the road system under stress. From a disaster risk reduction and civil protection perspective, the formation of isolated components is critical. Under major flood conditions, an urban mobility system can transition from a coherent, city-wide network into several segregated subnetworks with limited or no interconnection. Preparedness planning must therefore anticipate the temporary isolation of neighbourhoods and incorporate decentralised evacuation routes, distributed placement of emergency services, and redundancy in access to critical facilities within each potential subnetwork.

These findings demonstrate that river flooding drives system-wide transformations in urban road networks that extend well beyond the immediate spatial footprint of inundation. Non-linear losses of connectivity, shifts in node centrality, and the emergence of isolated subnetworks underline the necessity of adopting systemic, network-based approaches to flood risk assessment and disaster risk reduction [3,10,26,35]. Incorporating such insights into risk analysis allows for more accurate identification of vulnerable corridors and critical junctions, supports the prioritisation of investments in flood-resilient infrastructure, and informs evacuation strategies that explicitly anticipate the formation of isolated network components and functional “islands” of accessibility [36,37]. For stakeholders such as municipal planners, road and transport authorities, emergency management agencies, and operators of critical services (e.g., healthcare, fire, and rescue utilities), these results provide an evidence base for designing redundancy into key routes, pre-positioning emergency resources across multiple subnetworks, and coordinating cross-agency contingency planning. In this way, network-oriented flood impact analysis not only advances the understanding of physical exposure but also strengthens the decision-making processes that underpin urban resilience and civil protection.

This study models structural disruptions by removing inundated road segments from the network, thereby providing a first-order representation of flood-induced loss of connectivity. However, the approach does not yet account for dynamic traffic behaviour, temporal flood evolution, or spatial variations in flow velocity, all of which can substantially influence realised disruption and travel-time impacts. The current framework does not model traffic demand redistribution or load evolution on the post-flood network. Capturing congestion and travel-time impacts would require coupling the topological analysis with traffic assignment or agent-based mobility models, which constitutes a natural extension of this work. In addition, the use of simplified geometric representations of road segments may affect the precision with which inundation is detected at fine spatial scales. While these simplifications do not undermine the core conclusions regarding systemic vulnerability patterns, they delineate important avenues for further research, including the integration of time-varying hydrodynamics, refined road geometries, and dynamic mobility datasets. Additionally, the flood hazard layers assume a spatially uniform return period, implying simultaneous inundation of equal magnitude across the entire domain. Different flood events with the same return period can produce distinct spatial patterns, and the uniform assumption may overestimate the extent of concurrent disruption. This approach remains suitable for stress-testing the framework under conservative conditions, but future applications could incorporate spatially distributed or ensemble-based flood scenarios to capture a wider range of plausible disruption patterns.

The analytical framework applied in this study is scale-independent, meaning that it can be applied to road networks of different sizes and spatial extents, from small urban districts to large metropolitan areas. It relies on widely available data sources, namely road network geometry and flood hazard maps, making it transferable to many cities and urban contexts. The approach can be readily replicated in other areas to identify critical nodes, potentially disconnection zones, and priority regions for flood risk reductions. Although the specific patterns of fragmentation depend on local topography and network design, the underlying mechanism observed here—non-linear connectivity loss, the emergence of isolated subnetworks, and shifts in criticality—are likely to occur in other flood-prone areas as well. Thus, the study offers transferable insights for cities seeking to understand how hydrological hazards propagate through transportation systems and where systemic vulnerabilities may arise.

## 5. Conclusions

This study has presented a complex network–based framework for assessing how flooding alters the structural and functional organization of an urban road network. By integrating flood hazard information with graph-theoretical indicators, we show that inundation under the analyzed scenarios can generate non-linear and disproportionate impacts on connectivity, accessibility, and overall network cohesion. The 100-year flood scenario already induces noticeable fragmentation and a reduction in system-wide accessibility, whereas the extreme BHF event leads to the emergence of extensive critical junctions and partitions the network into several semi-isolated regions. These patterns are consistent with previous findings that localised flooding can trigger abrupt, system-wide disruptions in transportation systems [13].

The results further demonstrate that vulnerability in urban road networks is not determined solely by which links and nodes are physically inundated, but by how the entire system reorganises when key corridors and intersections are compromised. Nodes and links that are structurally important under normal conditions may lose influence during flooding, while previously peripheral elements can become critical as accessibility is rerouted and localised subnetworks emerge. By jointly applying degree, closeness, and betweenness centrality together with community separation analysis, this study fills an important gap in the literature: for the first time, these complementary metrics are integrated within a single framework to characterise flood impacts. This combined analysis provides deeper insight into the nonlinear reconfiguration of road networks under flood stress, revealing shifts in functional importance that cannot be captured by any single indicator alone.

Overall, the study underscores the value of network-based approaches as a complement to traditional flood risk assessments. By revealing the systemic consequences of flooding beyond the immediate inundation footprint, the proposed framework provides actionable insights for strengthening urban resilience, improving evacuation and emergency response planning, and supporting risk-informed spatial and infrastructural decision-making under current and future flood scenarios.
