# Supplementary material for: Non-linear failure patterns in urban road networks exposed to flooding
Source: PLoS One. 2026 Sep 1;21(9):e0354204. doi: 10.1371/journal.pone.0354204 (PMC13533346; doi:10.1371/journal.pone.0354204)
Supplement: Supplementary file 1 [file pone.0354204.s002.pdf]

Non-linear failure patterns in urban road networks exposed to flooding Ref.: PONE-D-25-67852

We would like to thank the reviewers for their constructive comments and suggestions. The resubmitted manuscript has undergone a thorough revision according to the reviewers' comments (changes in blue in the manuscript, red color indicates the answers to reviewers). Please see our responses below.

Reviewer 1

This study models road network disruptions from flood events by removing inundated road segments, thereby providing a first-order representation of flood-induced loss of connectivity. It presents an analytical framework that jointly captures how local connectivity, shortest-path structure, and mesoscale fragmentation evolve under inundation, employing degree analysis, closeness centrality, betweenness centrality, and network component analysis across flood scenarios. The inputs to the framework are the road network and the flood scenario, including its water depth. Three scenarios are analyzed: the baseline scenario and scenarios with probabilities of exceedance of 1% and 0.01%. In general, I found this study interesting and valuable, as it could provide insights into how a road network responds to a given flood event, offering stakeholders and emergency responders information on urban resilience and emergency response planning, integrating complementary metrics in a single framework. Its inputs and the mathematical representation made this framework feasible to apply to different study cases. However, after a careful review, I have some comments that I encourage the authors to address to clarify some points in the study.

Reply: We would like to thank reviewer for the thorough assessment of our manuscript. We have carefully reviewed all their comments and addressed them accordingly. Please find below our response.

Major suggested comments

Reviewer Comment 1 — 1.[Lines 91-92] In general, the use of a hazard layer as an inundation layer is not accurate, since the underlying assumption of the hazard layer is that all the reaches inside of the domain are being affected by a flow with the same return period, which is unfeasible, in this case, 100-year or 10,000-year. Intense storm events rarely cover the entire domain uniformly, creating this kind of scenario. Using the hazard layer in this case would exaggerate the potential effects on the road network. Also, it is important to note that different flood scenarios can have the same return period but different spatial patterns within the domain, resulting in different responses of the road system. [Lines 360-361] The mentioned above aligns with what is presented in these lines, since the configuration of the hazard and its high spatial variability highly influences this kind of analysis. For the purposes of this study, using this layer may be valid to illustrate the effect of flood extent, but I suggest including the limitation stated above.

Reply: We thank the reviewer for this insightful observation. We would like to clarify that the flood data used in this study are probabilistic hazard maps associated with specific return periods, produced through hydrodynamic modelling by the Swedish Civil Contingencies Agency (MSB). The spatial uniformity of the return period is inherent to these probabilistic products rather than a modelling choice made by the authors. We agree, however, that this assumption does not capture the spatial variability of real flood events, and that different inundation patterns could produce different network responses. We have revised the discussion in Section 4 to acknowledge this limitation explicitly, see [Lines 621-631] in the revised version:

"Additionally, the probabilistic flood hazard layers used in this study assign a uniform return period across the entire domain. While this is inherent to the statistical framework underpinning the hazard maps, it does not capture the spatial variability that characterises real flood events. Future applications could complement the probabilistic approach with ensemble-based or event-specific hydrodynamic simulations to explore how different inundation patterns for the same return period affect network responses".

Reviewer Comment 2 — [Lines 135-136] This sentence states the water depth threshold (20 cm) for determining whether an edge is flooded and should be removed from the network topology; however, the rationale supporting this value is not provided. It should be included, since this parameter governs all subsequent analysis.

Reply: We agree that the threshold used to classify flooded road segments requires explicit justification because it governs the subsequent network-topology analysis. In the revised manuscript, we have clarified that $h_k > 0.2m$ is adopted as an operational impassability criterion for regular passenger-vehicle traffic. This value is consistent with flood-transport studies indicating that relatively shallow inundation can already compromise vehicle movement, traction, and manoeuvrability. Reported disruption depths for passenger vehicles commonly fall within the range of approximately 0.15–0.30 m, depending on vehicle type, flow velocity, road surface, and driver behaviour [7,17]. The selected 0.2 m threshold therefore lies within this established disruption range and provides a conservative criterion for representing road segments as functionally unavailable under flood conditions. We have added this rationale and the corresponding references to the revised manuscript, see [Lines 221-230] in Section 2.3.2.

"A road segment k was classified as flooded and removed from the functional road network when the inundation depth exceeded $h_k > 0.2m$. This threshold was adopted as an operational impassability criterion for regular passenger-vehicle traffic. Previous flood-transport studies indicate that shallow inundation can compromise vehicle movement, traction, and manoeuvrability, with reported disruption depths for passenger vehicles commonly falling within approximately 0.15–0.30 m depending on vehicle type, flow velocity, road surface, and driver behaviour [7,17]. The use of 0.2 m therefore provides a conservative and literature-supported criterion for identifying road segments that are functionally unavailable during flooding."

Reviewer Comment 3 — [Lines 146-147] Be careful with the statement "most affected by flood-induced disruptions". As mentioned before, a real flood event will not affect the entire domain with the same intensity across all subregions (streets) within the city, which is the basis assumption of a flood hazard map. Also, a real flood extent will depend not only on the system configuration but also on the precipitation configuration. Different events lead to different flood extents. State that the most affected areas identified by a single event, based on the flood hazard map, are not

accurate. To identify the most affected, it is necessary to analyze multiple potential events in the domain and assess how often an area is affected.

Reply: As discussed in our response to Comment 1, the flood data are probabilistic about hazard maps rather than deterministic event simulations. We have revised the statement in Section 2.3.2 to clarify that the identified critical nodes correspond to the specific hazard level considered, and that different return periods may highlight different vulnerable locations, as follows, see [Lines 245-247] in the revised version:

"The comparison between the pre-flood and post-flood network configurations allows the identification of nodes and areas that are most affected under the specific probabilistic hazard level considered."

Reviewer Comment 4 — [Equation 6, 7, 8, 9] What is the purpose of introducing equations 6, 7, 8, and 9 if they are not used or mentioned afterward to characterize the modularity analysis of the system

Reply: In the revised manuscript, we have removed the numbering of the proposed equations. However, we have retained the equations, as they are necessary for defining the modularity index, which is also depicted in Figure 9 to illustrate the network dynamics.

Reviewer Comment 5 — [Equation 8] How does this equality hold? The (1+) part of the equation disappeared.

Reply: We agree with the reviewer that the previously labeled Equation (8) (new version eq.(6)) requires further justification. The detailed steps have now been provided and incorporated into the revised manuscript. The proof is based on network theory; specifically, the sum of all elements of the adjacency matrix A equals twice the total number of edges, i.e., $\sum_{ij} A_{ij} = \sum_j \sum_i A_{ij} = \sum_j k_j = 2m$. Then, eq.(6) is written

$$Q_2 = \frac{1}{4m} \sum_{i,j} \left(A_{ij} - \frac{k_i k_j}{2m} \right) (1 + s_i s_j) = \frac{1}{4m} \left[\sum_{i,j} \left(A_{ij} - \frac{k_i k_j}{2m} \right) + \sum_{i,j} \left(A_{ij} - \frac{k_i k_j}{2m} \right) s_i s_j \right].$$

The second part is equal to zero since

$$\sum_{i,j} \left(A_{ij} - \frac{k_i k_j}{2m} \right) = \sum_{i,j} A_{ij} - \sum_{i,j} -\frac{k_i k_j}{2m} = 2m - \frac{1}{2m} \sum_i k_i \sum_j k_j = 2m - \frac{1}{2m}(2m)^2 = 0$$

The following lines were added in eq.(6)(in the new version):

$$Q_2 = \frac{1}{4m} \sum_{i,j} \left(A_{ij} - \frac{k_i k_j}{2m} \right) (1 + s_i s_j) = \frac{1}{4m} \left[\sum_{i,j} \left(A_{ij} - \frac{k_i k_j}{2m} \right) + \sum_{i,j} \left(A_{ij} - \frac{k_i k_j}{2m} \right) s_i s_j \right].$$

The sum of all elements of the adjacency matrix A equals twice the total number of edges, i.e., $\sum_{ij} A_{ij} = \sum_j \sum_i A_{ij} = \sum_j k_j = 2m$, which implies that the first part is equal to zero. Then, eq.(6) is written

$$Q_2 = \sum_{i,j} \left(A_{ij} - \frac{k_i k_j}{2m} \right) s_i s_j = \frac{1}{4m} \mathbf{s}^T B \mathbf{s},$$

Reviewer Comment 6 — [Lines 234] In the baseline scenario, Fig. 4a, there are no isolated nodes; how could you affirm this?

Reply: In the baseline scenario, there are no nodes with zero degree. However, the network structure contains several small, isolated clusters. Specifically, there are 22 clusters consisting of two nodes connected by a single edge. In addition, there are three clusters of four nodes (with a tree structure), one cluster of six nodes, and one cluster of seven nodes. The remaining 2,588 nodes form a giant component.

The following sentence was added in the caption of Fig 4:

"The majority of nodes have degree one and three, while there are no nodes with zero degree".

The following sentences were added in section 3.4 (see [Lines 486-489] in the revised version) :

"Specifically, the network contains three major components comprising 716 (red), 744 (yellow), and 463 (purple) nodes, respectively (Fig. 9a). In addition, 263 smaller components have become mutually disconnected following the flooding of key bridging edges."

and (see [Lines 494-504] in the revised version) :

"Under the extreme BHF scenario (Fig. 9b), only four large components with more than 70 nodes remain, each corresponding to a major subregion of the city. At the same time, the number of small isolated components increases from 263 in the 100-year scenario to 553. Specifically, the network now contains three major components comprising 625 (red), 619 (yellow), and 465 (purple) nodes, respectively, as shown in Fig. 9b."

Reviewer Comment 7 — [Lines 235-237] What is the mean degree for scenario c? It could be useful to plot the mean degree in the figure and add the bar values to facilitate better comparison.

Reply: The mean degree under the extreme flood scenario (BHF) is 1.805. This value has now been included in Figure 4 and in Section 3.1 of the revised manuscript. In addition, red vertical lines indicating the mean values have been added to all panels of Figure 3 to facilitate comparison.

Reviewer Comment 8 — [Lines 240-241] According to equation (4), it is not just the inverse; it is the number of nodes j that can be reached from $i(N-1)$ over the cumulative shortest path to nodes j from i.

Reply: We have now improved the sentence mentioned by the reviewer accordingly, on new page 9 below equation.(6), see [Lines 299-300] in the revised version:

"....which represents the reciprocal of the average shortest path length from node i multiplied by the number of $N-1$ other nodes that can be reached from i."

Reviewer Comment 9 — [Lines 243- 244, Figure 5] Considering the definition above, the metric of closeness centrality is not upper-bounded by 1 (as it seems in Figure 5), that would happen only if the cumulative shortest path is equal to the number of nodes, it would mean that, from node i, it is possible to reach all nodes j in one step on average, which is not possible. Following this, the metric must be well below 1, unless you are showing a normalized metric in the figure for better plotting. If that is the case, please clarify it.

Reply: We thank the reviewer for highlighting this point. In all computations and for each scenario, closeness centrality values were normalized by dividing them by the corresponding maximum closeness centrality value. We have added this clarification on page 12 (in the revised version) and in the caption of Figure 5 as follows:
"In our computation, we normalized the closeness value by dividing it by the maximum value of the network configuration."

Reviewer Comment 10 — [Description figure 6] "nearly symmetric around its mean". What is the mean? If you mention it, it should be shown in all three scenarios.

Reply: We clarify that this sentence refers only to the no flood case, i.e., Fig 6a. In the revised Figure 6a, we included the mean value, which highlights the near symmetry (with respect to the mean) of the no-flood distribution. We added in the caption of Figure 6a the following sentence:
"In the baseline scenario, the distribution is nearly symmetric around its mean (vertical line mean=0.701)..."

Reviewer Comment 11 — [Line 316] "The degree of fragmentation increases further." How did you measure this?

Reply: We agree with the reviewer and have revised the manuscript to include data supporting this statement. Specifically, in the 100-year scenario, the network contains five large components, whereas in the BHF scenario, only four large components remain. In both scenarios, there are three main clusters; however, under the BHF scenario, the number of nodes is substantially reduced in two of these three clusters. In addition, the number of small isolated clusters, defined as clusters with fewer than 70 nodes, increases from 263 to 553. The number of isolated nodes, i.e., nodes with degree zero, also increases. Taken together, these results indicate that the intensity of network fragmentation increases further under the BHF scenario. This revision also addresses Question 12 regarding the supporting data. The revised paragraph in Section 3.4 now reads, see [Lines 486-499] in the revised version:

"Specifically, the network contains three major components comprising 716 (red), 744 (yellow), and 463 (purple) nodes, respectively (Fig. 9a). In addition, 263 smaller components have become mutually disconnected following the flooding of key bridging edges. The majority of these 263 smaller clusters are isolated nodes — shown in light green — mainly located near the river, indicating the fragmentation of local streets and secondary connections. Under the extreme BHF scenario (Fig. 9b), only four large components with more than 70 nodes remain, each corresponding to a major subregion of the city. At the same time, the number of small isolated components increases from 263 in the 100-year scenario to 553. Specifically, the network now contains three major components comprising 625 (red), 619 (yellow), and 465 (purple) nodes, respectively, as shown in Fig. 9b. This indicates a substantial reduction in the number of nodes in the major components compared with the 100-year scenario. Considering both the increase in isolated nodes, defined as nodes with degree zero (Fig. 4c), and the rise in the number of isolated clusters from 264 to 553, we conclude that network fragmentation becomes more severe under the BHF scenario. The light green cluster of isolated nodes becomes more pronounced (compared to the 100-year case), reflecting widespread disconnection of local roads, especially in low-lying and peripheral areas."

Reviewer Comment 12 — [Lines 317-320] This analysis could be supported by data, e.g. number of nodes in each cluster.

Reply: Please look at the previous answer of comment 11, where data is provided.

Reviewer Comment 13 — [Line 350] "The original network exhibits a coarse partitioning into three main subnetworks". By visual inspection, this is not noticeable. How did you conclude that?

Reply: The original sentence was inaccurate: the three-subregion partition refers to the 100-year flood scenario (Fig. 5b), not to the baseline network (Fig. 5a). In Fig. 5b, the flood appears to divide the network, at a coarse spatial scale, into three areas: east, west, and south of the river. This separation persists under the more intense BHF scenario and is further supported by the component analysis, which identifies three major components in Section 3.4. We have corrected the sentence (in the new version [Lines 551-556]) as follows:
"As shown in Figs. 5b and 5c, the structure of the initial network changes substantially under flooding. The flooded river broadly divides the city network into three main areas: east, west, and south of the river. The red regions in Fig. 5b indicate the epicentres of these subnetworks. This spatial separation persists under the more intense BHF scenario (Fig. 5c). The same pattern is further supported by the component analysis, which identifies three major components."

Reviewer Comment 14 — [Line 407] "relatively moderate inundation" 100 yr return period is not a "relatively moderate inundation", nor is the BHF. No more scenarios were tested to support this conclusion.

Reply: We agree that the term "moderate" was inaccurate for describing a 100-year return period flood. We have revised the manuscript to remove this term and instead contrast the 100-year scenario against the extreme BHF event, clarifying that significant network disruption already occurs under the 100-year flood without requiring extreme magnitudes. We changed the whole phrase accordingly, please see in the revised version [Lines 650-655]):
"By integrating flood hazard information with graph-theoretical indicators, we show that inundation under the analyzed scenarios can generate non-linear and disproportionate impacts on connectivity, accessibility, and overall network cohesion. The 100-year flood scenario already induces noticeable fragmentation and a reduction in system-wide accessibility, whereas the extreme BHF event leads to the emergence of extensive critical junctions and partitions the network into several semi-isolated regions."

Reviewer Comment 15 — [Lines 410-412] It could be useful to see the flooded areas to support the statement of "small flooded areas", or drop a metric like % of area flooded.

Reply: We thank the reviewer for this observation. The phrase "small flooded areas" referred to a general finding from the cited literature rather than a metric derived from our own data. To avoid ambiguity, we have replaced it with "localized flooding," ([Lines 657-659], revised version) which accurately reflects the claim being made without implying a specific quantitative characterization of our study scenarios.

Minor suggested comments

Reviewer Comment 16 — [Figure 1] It is supposed, according to the text, to show the location of the city, highlighting the Klarälven River and the Lake Vänern; however, the figure is big, and it is not clear about the study area, since it is not delimited.

Reply: We thank the reviewer for this helpful suggestion. Figure 1 has been completely redesigned to clearly delineate the study area, label the Klarälven River and Lake Vänern, and include an inset showing Karlstad's location within Sweden. In the revised version, Figure 1 now contains two maps of the study area corresponding to the two flood scenarios considered in this study, namely the 100-year flood and the BHF scenario. These scenarios are shown using distinct colours to improve readability and to provide immediate visual context for the subsequent network analysis. This revised figure more clearly identifies the spatial extent of the study area and its relationship to the main hydrological features relevant to the flood-risk assessment.

Reviewer Comment 17 — [Description Figure 5, line 6] "Broadly similar", like what? Scenario b?

Reply: We thank the reviewer for this observation. "Broadly similar" was intended as a reference to the 100-year flood scenario (b). We have revised the caption to state this explicitly, replacing "broadly similar" with "broadly similar to the 100-year flood scenario (b)" to remove the ambiguity.

Reviewer Comment 18 — [Lines 273-274] It is worth putting the mean shortest path length for each scenario. It would allow a better comparison.

Reply: We followed the reviewer's suggestion. Initially, we dealt with the existence of many disconnected components. Then, $d_{ij} = +\infty$ if i and j belong to different components. To overcome this problem, we utilize the global efficiency, which is defined as

$$E_{\text{glob}} = \frac{1}{N(N-1)} \sum_{i \neq j} \frac{1}{d_{i \to j}} \tag{1}$$

As an alternative to the mean path length, we define

$$\overline{m}_2 = \frac{1}{E} \tag{2}$$

Using $\overline{m}_2$, we obtain the following results: baseline $\overline{m}_2 = 37.7$, 100-year flood $\overline{m}_2 = 81.64$, and BHF $\overline{m}_2 = 102.08$. This breakdown better illustrates the progressive decline in routing efficiency across flood scenarios. In the updated version, the definition of the global efficiency is provided in Section 2.3.3, see equation (3), and at the end of Section 3.2 on page 12 [Lines 425-428].
"The mean shortest path length $\overline{m}_2$ defined from eq.(3) increases across scenarios, from $\overline{m}_2 = 37.7$ baseline to $\overline{m}_2 = 81.64$ in the 100-year flood, and $\overline{m}_2 = 102.08$ in BHF, reflecting a progressive deterioration in routing efficiency as inundation expands."

Reviewer Comment 19 — 4. [Figure 8] Use the same $x-$axis limits for better comparison between the scenarios.

Reply: We tried the reviewer's suggestion; however, we decided not to include it for the following reason: For the 100-year flood and BHF scenarios, betweenness centrality values are one order of magnitude smaller than in the no-flood case. As a result, almost the entire distribution falls within the first histogram bin of no flood cases, obscuring rather than revealing meaningful differences.

Reviewer Comment 20 — [Figure 8a] Axis labels for Figure 8a are different from those of the other figures. It is possible to include the power-law distribution line and its equation in the graphs.

Reply: We thank the reviewer for the suggestion, and in the revised paper, we included the power-law distribution in the histograms. Furthermore, as a separate inset, we present the power law on a logarithmic scale to highlight the differences among the three cases.

Reviewer Comment 21 — [Figure 9] Using the same colors for the same clusters for both scenarios would allow a better comparison.

Reply: We thank the reviewer for this suggestion. Figure 9 has been revised so that corresponding clusters share consistent colors across both flood scenarios, allowing direct visual comparison of how spatial components evolve between the 100-year and BHF scenarios.

Reviewer 2

This paper proposes a scale-independent analytical framework to measure the evolution of road network performance under inundation conditions. The following suggestions aim to improve the clarity and rigor of the manuscript.

Reply: We would like to thank reviewer for the thorough assessment of our manuscript. We have carefully reviewed all their comments and addressed them accordingly. Please find below our response.

Reviewer Comment 1 — The novelty and contribution of the paper should be more clearly articulated. Specifically, it is necessary to justify why the proposed indicators, local connectivity, shortest path structure, and meso-scale fragmentation, are essential for capturing network evolution. Given that existing studies have extensively evaluated the dynamic vulnerability, resilience, and robustness of road networks during flooding, the authors should explicitly highlight what distinguishes this scale-independent framework from prior work.

Reply: We agree that the novelty and contribution of the paper should be stated more explicitly. In the revised manuscript, we have therefore clarified that the novelty of this work does not lie in proposing entirely new network metrics, since degree, closeness centrality, betweenness centrality, and community detection are well-established tools in network science. Rather, the contribution lies in integrating these indicators into a unified, scale-independent framework specifically designed to capture how flood-induced disruptions propagate through an urban road network across different structural levels.

This distinction is important because existing studies on flood-related road network vulnerability, resilience, and robustness often focus on overall performance loss, accessibility reduction, or critical-link identification. While such approaches are valuable, they may not fully explain how the internal structure of the network evolves during flooding. Our framework addresses this gap by combining indicators that capture complementary dimensions of network degradation: local connectivity reflects the loss of immediate redundancy around affected nodes; shortest-path-based indicators reveal the reorganization of accessibility and traffic flow routes; betweenness centrality identifies the emergence or disappearance of critical transfer corridors; and meso-scale fragmentation captures how the network separates into increasingly isolated communities.

By considering these indicators together, the proposed framework makes it possible to identify non-linear failure dynamics and structural transitions that would remain partially hidden if each metric were analyzed in isolation. In particular, it allows us to distinguish between localized disruptions, network-wide accessibility decline, concentration of movement on a limited number of remaining routes, and the fragmentation of the system into disconnected or weakly connected areas. This combined perspective provides a more spherical understanding of flood impacts on road networks and supports more targeted mitigation and planning strategies for municipalities, including the identification of critical areas, infrastructure reinforcement priorities, emergency access routes, and evacuation planning.

We have revised the manuscript accordingly to explicitly justify why local connectivity, shortest-path structure, and meso-scale fragmentation are essential for capturing network evolution under flooding, and to better distinguish the proposed scale-independent framework from previous studies. The corresponding revisions can be found in the revised manuscript at lines [115–133], as follows:

"Despite these advances, existing approaches tend to emphasise either static topological metrics [11,13] or aggregate network-connectivity indicators [12,14], each providing only a partial view of how failures propagate through the system. Centrality-based studies quantify shifts in node importance but do not fully characterise how the network separates into functionally distinct subregions. Similarly, global connectivity-based approaches capture overall changes in network cohesion but provide limited insight into how accessibility and flow patterns reorganise at the node and meso-scale levels. To date, these complementary perspectives have rarely been integrated within a unified analytical framework for assessing flood-induced road-network evolution. The present work addresses this gap by jointly tracking degree, closeness, and betweenness centrality together with community-structure analysis across flood scenarios. This combination is essential because each metric captures a distinct dimension of network degradation: degree reflects the loss of local redundancy, closeness reveals the reorganisation of accessibility, betweenness identifies emerging bottlenecks and flow concentration, and community detection exposes meso-scale fragmentation and the formation of isolated subnetworks. Analysed jointly, these indicators reveal non-linear failure dynamics, such as the simultaneous emergence of uneven accessibility patterns and the concentration of connectivity onto critical corridors, which cannot be detected by any single metric applied in isolation. "

Reviewer Comment 2 — To evaluate the indirect impacts of road network after flooding, there are no simulation or data about the load evolution on road network.

Reply: We thank the reviewer for this observation. We acknowledge that the current framework focuses on the structural and topological response of the road network and does not incorporate traffic demand data or simulate load redistribution onto remaining links. Modelling indirect impacts such as congestion propagation and travel-time increases would require coupling the network analysis with traffic assignment or agent-based simulation models, which falls beyond the scope of this study. We have added this as an explicit limitation in Section 4 and identified it as a direction for future research. We believe, however, that the topological analysis presented here provides a necessary structural foundation for such dynamic extensions, as it identifies the critical nodes, bottlenecks, and isolated subnetworks around which traffic redistribution would occur. Our response to this comment can be found in the revised version at lines [612–616] as follows:

"The current framework does not model traffic demand redistribution or load evolution on the post-flood network. Capturing congestion and travel-time impacts would require coupling the topological analysis with traffic assignment or agent-based mobility models, which constitutes a natural extension of this work."

Reviewer Comment 3 — The term "moderate flooding" should be clearly defined. Additionally, the threshold setting of $h_k > 0.2$ m requires a solid justification or reference to established standards or previous studies to support its validity.

Reply: We agree that the term "moderate" was inaccurate for describing a 100-year return period flood. We have revised the manuscript to remove this term and instead contrast the 100-year scenario against the extreme BHF event, clarifying that significant network disruption already occurs under the 100-year flood without requiring extreme magnitudes.

Regarding the 20 cm threshold, we agree that the threshold used to classify flooded road segments requires explicit justification because it governs the subsequent network-topology analysis. In the revised manuscript, we have clarified that $h_k > 0.2m$ is adopted as an operational impassability criterion for regular passenger-vehicle traffic. This value is consistent with flood-transport studies indicating that relatively shallow inundation can already compromise vehicle movement, traction, and manoeuvrability. Reported disruption depths for passenger vehicles commonly fall within the range of approximately 0.15–0.30 m, depending on vehicle type, flow velocity, road surface, and driver behaviour [7,17]. The selected 0.2 m threshold therefore lies within this established disruption range and provides a conservative criterion for representing road segments as functionally unavailable under flood conditions. We have added this rationale and the corresponding references to the revised manuscript, see [Lines 221-230] in Section 2.3.2.

"A road segment k was classified as flooded and removed from the functional road network when the inundation depth exceeded $h_k > 0.2m$. This threshold was adopted as an operational impassability criterion for regular passenger-vehicle traffic. Previous flood-transport studies indicate that shallow inundation can compromise vehicle movement, traction, and manoeuvrability, with reported disruption depths for passenger vehicles commonly falling within approximately 0.15–0.30 m depending on vehicle type, flow velocity, road surface, and driver behaviour [7,17]. The use of 0.2 m therefore provides a conservative and literature-supported criterion for identifying road segments that are functionally unavailable during flooding."

Reviewer Comment 4 — The results presented in the abstract should be more specific and quantitative. it is recommended to streamline this part and focus on the key findings and implications.

Reply: We thank the reviewer for this suggestion. We have revised the abstract to include specific quantitative results, including changes in mean degree, the shift in closeness centrality distribution, the steepening of the betweenness centrality power-law exponent, and the number of disconnected network components under each scenario. The abstract has also been streamlined to focus on key findings and their implications, see our response in [Lines 34-48] of the revised manuscript as well as below:

"Our analysis shows that inundation under the two flood scenarios can generate non-linear and disproportionate impacts on connectivity, accessibility, and overall network cohesion. Specifically, by examining extreme flood events for the 100-year and BHF scenarios, our method: (a) localizes critically isolated nodes exposed to high risk; (b) identifies structural changes, showing that the flooded network is divided into three main large subnetworks with specific epicenters, along with 263 smaller subnetworks in the 100-year case and 553 in the BHF case; (c) estimates that network inefficiency, interpreted as analogous to mean travel time to a destination, increases by 120% and 170% for the 100-year and BHF scenarios, respectively; and (d) localizes critical roads that may serve as potential corridors during evacuation planning. These results offer actionable insights for disaster risk reduction, including

planning for neighborhood-scale isolation, identifying vulnerable corridors, and designing redundant and decentralized emergency access routes. The framework is transferable to other urban areas where flood maps and road network data are available, supporting risk-informed spatial planning and strengthened civil protection."
