# Supplementary material for: Non-linear failure patterns in urban road networks exposed to flooding
Source: PLoS One. 2026 Sep 1;21(9):e0354204. doi: 10.1371/journal.pone.0354204 (PMC13533346; doi:10.1371/journal.pone.0354204)
Supplement: Peer review history

# Peer review: Decision Letter 0

**DOI:** 10.1371/journal.pone.0354204.r001

**Authors:** Qing-Chang Lu

26 Mar 2026

Dear Dr. Spiliotis,

Thank you for submitting your manuscript to PLOS ONE. After careful consideration, we feel that it has merit but does not fully meet PLOS ONE’s publication criteria as it currently stands. Therefore, we invite you to submit a revised version of the manuscript that addresses the points raised during the review process.

Please submit your revised manuscript by May 10 2026 11:59PM. If you will need more time than this to complete your revisions, please reply to this message or contact the journal office at plosone@plos.org . When you're ready to submit your revision, log on to https://www.editorialmanager.com/pone/ and select the 'Submissions Needing Revision' folder to locate your manuscript file.

- A letter that responds to each point raised by the academic editor and reviewer(s). You should upload this letter as a separate file labeled 'Response to Reviewers'.
- A marked-up copy of your manuscript that highlights changes made to the original version. You should upload this as a separate file labeled 'Revised Manuscript with Track Changes'.
- An unmarked version of your revised paper without tracked changes. You should upload this as a separate file labeled 'Manuscript'.

If you would like to make changes to your financial disclosure, please include your updated statement in your cover letter. Guidelines for resubmitting your figure files are available below the reviewer comments at the end of this letter.

If applicable, we recommend that you deposit your laboratory protocols in protocols.io to enhance the reproducibility of your results. Protocols.io assigns your protocol its own identifier (DOI) so that it can be cited independently in the future. For instructions see: https://journals.plos.org/plosone/s/submission-guidelines#loc-laboratory-protocols . Additionally, PLOS ONE offers an option for publishing peer-reviewed Lab Protocol articles, which describe protocols hosted on protocols.io. Read more information on sharing protocols at https://plos.org/protocols?utm_medium=editorial-email&utm_source=authorletters&utm_campaign=protocols .

As the corresponding author, your ORCID iD is verified in the submission system and will appear in the published article. PLOS supports the use of ORCID, and we encourage all coauthors to register for an ORCID iD and use it as well. Please encourage your coauthors to verify their ORCID iD within the submission system before final acceptance, as unverified ORCID iDs will not appear in the published article. *Only*  the individual author can complete the verification step; PLOS staff *cannot*  verify ORCID iDs on behalf of authors.

We look forward to receiving your revised manuscript.

Kind regards,

Qing-Chang Lu

Academic Editor

PLOS One

Journal Requirements:

When submitting your revision, we need you to address these additional requirements.

1. Please ensure that your manuscript meets PLOS ONE's style requirements, including those for file naming. The PLOS ONE style templates can be found at

https://journals.plos.org/plosone/s/file?id=wjVg/PLOSOne_formatting_sample_main_body.pdf and

https://journals.plos.org/plosone/s/file?id=ba62/PLOSOne_formatting_sample_title_authors_affiliations.pdf .

2. Thank you for stating the following financial disclosure:

“1) Swedish Research Council for Sustainable Development (FORMAS) under Grant Nos. 2021-02380-3 and 2021-02388-8.

2) Societal Risk Research (CSR) , Karlstad University”

Please state what role the funders took in the study.  If the funders had no role, please state: "The funders had no role in study design, data collection and analysis, decision to publish, or preparation of the manuscript."

If this statement is not correct you must amend it as needed.

Please include this amended Role of Funder statement in your cover letter; we will change the online submission form on your behalf.

3. PLOS requires an ORCID iD for the corresponding author in Editorial Manager on papers submitted after December 6th, 2016. Please ensure that you have an ORCID iD and that it is validated in Editorial Manager. To do this, go to ‘Update my Information’ (in the upper left-hand corner of the main menu), and click on the Fetch/Validate link next to the ORCID field. This will take you to the ORCID site and allow you to create a new iD or authenticate a pre-existing iD in Editorial Manager.

4. Please update your submission to use the PLOS LaTeX template. The template and more information on our requirements for LaTeX submissions can be found at http://journals.plos.org/plosone/s/latex .

5. Thank you for stating the following in the Acknowledgments Section of your manuscript:

“Konstantinos Karagiorgos acknowledges funding from the Swedish Research Council for Sustainable Development 429 (FORMAS) under Grant Nos. 2021-02380-3 and 2021-02388-8. 430 Nikos I. Kavallaris acknowledges funding from Centre for Societal Risk Research (CSR) , Karlstad University”

We note that you have provided additional information within the Acknowledgements Section that is not currently declared in your Funding Statement. Please note that funding information should not appear in the Acknowledgments section or other areas of your manuscript. We will only publish funding information present in the Funding Statement section of the online submission form.

Please remove any funding-related text from the manuscript and let us know how you would like to update your Funding Statement. Currently, your Funding Statement reads as follows:

“1) Swedish Research Council for Sustainable Development (FORMAS) under Grant Nos. 2021-02380-3 and 2021-02388-8.

2) Societal Risk Research (CSR) , Karlstad University”

Please include your amended statements within your cover letter; we will change the online submission form on your behalf.

6. We note that Figure 1 in your submission contain [map/satellite] images which may be copyrighted. All PLOS content is published under the Creative Commons Attribution License (CC BY 4.0), which means that the manuscript, images, and Supporting Information files will be freely available online, and any third party is permitted to access, download, copy, distribute, and use these materials in any way, even commercially, with proper attribution. For these reasons, we cannot publish previously copyrighted maps or satellite images created using proprietary data, such as Google software (Google Maps, Street View, and Earth). For more information, see our copyright guidelines: http://journals.plos.org/plosone/s/licenses-and-copyright.

We require you to either (1) present written permission from the copyright holder to publish these figures specifically under the CC BY 4.0 license, or (2) remove the figures from your submission:

a. You may seek permission from the original copyright holder of Figure 1 to publish the content specifically under the CC BY 4.0 license.

We recommend that you contact the original copyright holder with the Content Permission Form (http://journals.plos.org/plosone/s/file?id=7c09/content-permission-form.pdf) and the following text:

“I request permission for the open-access journal PLOS ONE to publish XXX under the Creative Commons Attribution License (CCAL) CC BY 4.0 (http://creativecommons.org/licenses/by/4.0/). Please be aware that this license allows unrestricted use and distribution, even commercially, by third parties. Please reply and provide explicit written permission to publish XXX under a CC BY license and complete the attached form.”

Please upload the completed Content Permission Form or other proof of granted permissions as an "Other" file with your submission.

In the figure caption of the copyrighted figure, please include the following text: “Reprinted from [ref] under a CC BY license, with permission from [name of publisher], original copyright [original copyright year].”

b. If you are unable to obtain permission from the original copyright holder to publish these figures under the CC BY 4.0 license or if the copyright holder’s requirements are incompatible with the CC BY 4.0 license, please either i) remove the figure or ii) supply a replacement figure that complies with the CC BY 4.0 license. Please check copyright information on all replacement figures and update the figure caption with source information. If applicable, please specify in the figure caption text when a figure is similar but not identical to the original image and is therefore for illustrative purposes only.

The following resources for replacing copyrighted map figures may be helpful:

USGS National Map Viewer (public domain): http://viewer.nationalmap.gov/viewer/

The Gateway to Astronaut Photography of Earth (public domain): http://eol.jsc.nasa.gov/sseop/clickmap/

Maps at the CIA (public domain): https://www.cia.gov/library/publications/the-world-factbook/index.html and https://www.cia.gov/library/publications/cia-maps-publications/index.html

NASA Earth Observatory (public domain): http://earthobservatory.nasa.gov/

Landsat: http://landsat.visibleearth.nasa.gov/

USGS EROS (Earth Resources Observatory and Science (EROS) Center) (public domain): http://eros.usgs.gov/#

Natural Earth (public domain): http://www.naturalearthdata.com/

7. If the reviewer comments include a recommendation to cite specific previously published works, please review and evaluate these publications to determine whether they are relevant and should be cited. There is no requirement to cite these works unless the editor has indicated otherwise.

[Note: HTML markup is below. Please do not edit.]

Reviewers' comments:

Reviewer's Responses to Questions

**Comments to the Author**

1. Is the manuscript technically sound, and do the data support the conclusions?

Reviewer #1: Yes

Reviewer #2: Yes

**********

2. Has the statistical analysis been performed appropriately and rigorously?

Reviewer #1: Yes

Reviewer #2: Yes

**********

3. Have the authors made all data underlying the findings in their manuscript fully available?

The PLOS Data policy

Reviewer #1: No

Reviewer #2: Yes

**********

4. Is the manuscript presented in an intelligible fashion and written in standard English?

Reviewer #1: Yes

Reviewer #2: Yes

**********

Reviewer #1: Overall review comment:

This study models road network disruptions from flood events by removing inundated road segments, thereby providing a first-order representation of flood-induced loss of connectivity. It presents an analytical framework that jointly captures how local connectivity, shortest-path structure, and mesoscale fragmentation evolve under inundation, employing degree analysis, closeness centrality, betweenness centrality, and network component analysis across flood scenarios. The inputs to the framework are the road network and the flood scenario, including its water depth. Three scenarios are analyzed: the baseline scenario and scenarios with probabilities of exceedance of 1% and 0.01%.

In general, I found this study interesting and valuable, as it could provide insights into how a road network responds to a given flood event, offering stakeholders and emergency responders information on urban resilience and emergency response planning, integrating complementary metrics in a single framework. Its inputs and the mathematical representation made this framework feasible to apply to different study cases. However, after a careful review, I have some comments that I encourage the authors to address to clarify some points in the study.

Major suggested comments:

1. [Lines 91-92] In general, the use of a hazard layer as an inundation layer is not accurate, since the underlying assumption of the hazard layer is that all the reaches inside of the domain are being affected by a flow with the same return period, which is unfeasible, in this case, 100-year or 10,000-year. Intense storm events rarely cover the entire domain uniformly, creating this kind of scenario. Using the hazard layer in this case would exaggerate the potential effects on the road network. Also, it is important to note that different flood scenarios can have the same return period but different spatial patterns within the domain, resulting in different responses of the road system. [Lines 360-361] The mentioned above aligns with what is presented in these lines, since the configuration of the hazard and its high spatial variability highly influences this kind of analysis. For the purposes of this study, using this layer may be valid to illustrate the effect of flood extent, but I suggest including the limitation stated above.

2. [Lines 135-136] This sentence states the water depth threshold (20 cm) for determining whether an edge is flooded and should be removed from the network topology; however, the rationale supporting this value is not provided. It should be included, since this parameter governs all subsequent analysis.

3. [Lines 146-147] Be careful with the statement "most affected by flood-induced disruptions”. As mentioned before, a real flood event will not affect the entire domain with the same intensity across all subregions (streets) within the city, which is the basis assumption of a flood hazard map. Also, a real flood extent will depend not only on the system configuration but also on the precipitation configuration. Different events lead to different flood extents. State that the most affected areas identified by a single event, based on the flood hazard map, are not accurate. To identify the most affected, it is necessary to analyze multiple potential events in the domain and assess how often an area is affected.

4. [Equation 6, 7, 8, 9] What is the purpose of introducing equations 6, 7, 8, and 9 if they are not used or mentioned afterward to characterize the modularity analysis of the system

5. [Equation 8] How does this equality hold? The (1+) part of the equation disappeared.

6. [Lines 234] In the baseline scenario, Fig. 4a, there are no isolated nodes; how could you affirm this?

7. [Lines 235-237] What is the mean degree for scenario c? It could be useful to plot the mean degree in the figure and add the bar values to facilitate better comparison.

8. [Lines 240-241] According to equation 4, it is not just the inverse; it is the number of nodes j that can be reached from i (N-1) over the cumulative shortest path to nodes j from i.

9. [Lines 243- 244, Figure 5] Considering the definition above, the metric of closeness centrality is not upper-bounded by 1 (as it seems in Figure 5), that would happen only if the cumulative shortest path is equal to the number of nodes, it would mean that, from node i, it is possible to reach all nodes j in one step on average, which is not possible. Following this, the metric must be well below 1, unless you are showing a normalized metric in the figure for better plotting. If that is the case, please clarify it.

10. [Description figure 6] “nearly symmetric around its mean”. What is the mean? If you mention it, it should be shown in all three scenarios.

11. [Line 316] “The degree of fragmentation increases further.” How did you measure this?

12. [Lines 317-320] This analysis could be supported by data, e.g. number of nodes in each cluster.

13. [Line 350] “The original network exhibits a coarse partitioning into three main subnetworks”. By visual inspection, this is not noticeable. How did you conclude that?

14. [Line 407] “relatively moderate inundation” 100 yr return period is not a "relatively moderate inundation", nor is the BHF. No more scenarios were tested to support this conclusion.

15. [Lines 410-412] It could be useful to see the flooded areas to support the statement of "small flooded areas", or drop a metric like % of area flooded.

Minor suggested comments:

1. [Figure 1] It is supposed, according to the text, to show the location of the city, highlighting the Klarälven River and the Lake Vänern; however, the figure is big, and it is not clear about the study area, since it is not delimited.

2. [Description Figure 5, line 6] “Broadly similar”, like what? Scenario b?

3. [Lines 273-274] It is worth putting the mean shortest path length for each scenario. It would allow a better comparison.

4. [Figure 8] Use the same x-axis limits for better comparison between the scenarios.

5. [Figure 8a] Axis labels for Figure 8a are different from those of the other figures. It is possible to include the power-law distribution line and its equation in the graphs.

6. [Figure 9] Using the same colors for the same clusters for both scenarios would allow a better comparison.

Reviewer #2: This paper proposes a scale-independent analytical framework to measure the evolution of road network performance under inundation conditions. The following suggestions aim to improve the clarity and rigor of the manuscript:

(1) The novelty and contribution of the paper should be more clearly articulated. Specifically, it is necessary to justify why the proposed indicators, local connectivity, shortest path structure, and meso-scale fragmentation, are essential for capturing network evolution. Given that existing studies have extensively evaluated the dynamic vulnerability, resilience, and robustness of road networks during flooding, the authors should explicitly highlight what distinguishes this scale-independent framework from prior work.

(2) To evaluate the indirect impacts of road network after flooding, there are no simulation or data about the load evolution on road network.

(3) The term "moderate flooding" should be clearly defined. Additionally, the threshold setting of hk > 0.2 m requires a solid justification or reference to established standards or previous studies to support its validity.

(4) The results presented in the abstract should be more specific and quantitative. it is recommended to streamline this part and focus on the key findings and implications.

**********

what does this mean? ). If published, this will include your full peer review and any attached files.

If you choose “no”, your identity will remain anonymous but your review may still be made public.

**Do you want your identity to be public for this peer review?**  For information about this choice, including consent withdrawal, please see our Privacy Policy

Reviewer #1: No

Reviewer #2: No

**********

[NOTE: If reviewer comments were submitted as an attachment file, they will be attached to this email and accessible via the submission site. Please log into your account, locate the manuscript record, and check for the action link "View Attachments". If this link does not appear, there are no attachment files.]

To ensure your figures meet our technical requirements, please review our figure guidelines: https://journals.plos.org/plosone/s/figures

You may also use PLOS’s free figure tool, NAAS, to help you prepare publication quality figures: https://journals.plos.org/plosone/s/figures#loc-tools-for-figure-preparation.

NAAS will assess whether your figures meet our technical requirements by comparing each figure against our figure specifications.

# Author comment: Author response to Decision Letter 1

**DOI:** 10.1371/journal.pone.0354204.r002

15 May 2026

We would like to sincerely thank the Academic Editor and the reviewers for their careful evaluation of our manuscript, entitled “Non-linear failure patterns in urban road networks exposed to flooding,” authored by Konstantinos Karagiorgos, Nikos Kavallaris, Ole Sönnerborn, Fotis Maris, and Konstantinos Spiliotis. We are grateful for the constructive comments and valuable suggestions, which have helped us improve the clarity, structure, methodological presentation, and overall contribution of the paper.

Please find the detailed answers in the attached document.

Attachment Submitted filename: review_letter_answers.pdf

# Peer review: Decision Letter 1

**DOI:** 10.1371/journal.pone.0354204.r003

**Authors:** Qing-Chang Lu

6 Jul 2026

Non-linear failure patterns in urban road networks exposed to flooding

PONE-D-25-67852R1

Dear Dr. Spiliotis,

We’re pleased to inform you that your manuscript has been judged scientifically suitable for publication and will be formally accepted for publication once it meets all outstanding technical requirements.

Within one week, you’ll receive an e-mail detailing the required amendments. When these have been addressed, you’ll receive a formal acceptance letter and your manuscript will be scheduled for publication.

An invoice will be generated when your article is formally accepted. Please note, if your institution has a publishing partnership with PLOS and your article meets the relevant criteria, all or part of your publication costs will be covered. Please make sure your user information is up-to-date by logging into Editorial Manager at Editorial Manager® and clicking the ‘Update My Information' link at the top of the page. For questions related to billing, please contact billing support .

If your institution or institutions have a press office, please notify them about your upcoming paper to help maximize its impact. If they’ll be preparing press materials, please inform our press team as soon as possible -- no later than 48 hours after receiving the formal acceptance. Your manuscript will remain under strict press embargo until 2 pm Eastern Time on the date of publication. For more information, please contact onepress@plos.org.

Kind regards,

Qing-Chang Lu

Academic Editor

PLOS One

Additional Editor Comments (optional):

Reviewers' comments:

Reviewer's Responses to Questions

**Comments to the Author**

Reviewer #1: All comments have been addressed

Reviewer #2: All comments have been addressed

**********

2. Is the manuscript technically sound, and do the data support the conclusions?

Reviewer #1: Yes

Reviewer #2: Yes

**********

3. Has the statistical analysis been performed appropriately and rigorously?

Reviewer #1: Yes

Reviewer #2: Yes

**********

4. Have the authors made all data underlying the findings in their manuscript fully available?

The PLOS Data policy

Reviewer #1: Yes

Reviewer #2: Yes

**********

5. Is the manuscript presented in an intelligible fashion and written in standard English?

Reviewer #1: Yes

Reviewer #2: Yes

**********

Reviewer #1: The authors have thoroughly and satisfactorily addressed all concerns raised in my previous review. The manuscript has been significantly improved, and I have no further concerns

Reviewer #2: I have reviewed the authors' responses and revisions. All the issues I raised have now been fully resolved.

**********

what does this mean? ). If published, this will include your full peer review and any attached files.

If you choose “no”, your identity will remain anonymous but your review may still be made public.

**Do you want your identity to be public for this peer review?**  For information about this choice, including consent withdrawal, please see our Privacy Policy

Reviewer #1: No

Reviewer #2: No

**********

# Editor report: Acceptance letter

**DOI:** 10.1371/journal.pone.0354204.r004

**Authors:** Qing-Chang Lu

PONE-D-25-67852R1

PLOS One

Dear Dr. Spiliotis,

I'm pleased to inform you that your manuscript has been deemed suitable for publication in PLOS One. Congratulations! Your manuscript is now being handed over to our production team.

At this stage, our production department will prepare your paper for publication. This includes ensuring the following:

* All references, tables, and figures are properly cited

* All relevant supporting information is included in the manuscript submission,

* There are no issues that prevent the paper from being properly typeset

You will receive further instructions from the production team, including instructions on how to review your proof when it is ready. Please keep in mind that we are working through a large volume of accepted articles, so please give us a few days to review your paper and let you know the next and final steps.

Lastly, if your institution or institutions have a press office, please let them know about your upcoming paper now to help maximize its impact. If they'll be preparing press materials, please inform our press team within the next 48 hours. Your manuscript will remain under strict press embargo until 2 pm Eastern Time on the date of publication. For more information, please contact onepress@plos.org.

You will receive an invoice from PLOS for your publication fee after your manuscript has reached the completed accept phase. If you receive an email requesting payment before acceptance or for any other service, this may be a phishing scheme. Learn how to identify phishing emails and protect your accounts at https://explore.plos.org/phishing.

If we can help with anything else, please email us at customercare@plos.org.

Thank you for submitting your work to PLOS One and supporting open access.

Kind regards,

PLOS ONE Editorial Office Staff

on behalf of

Dr. Qing-Chang Lu

Academic Editor

PLOS One
